# Optimal DG allocation using the Dingo Optimization Algorithm: robust power loss reduction with concomitant voltage stability improvement in distribution and transmission networks

**DOI:** 10.1038/s41598-026-54963-6

**Published:** 2026-06-03

**Authors:** Hossam Kotb, George Michael, Kareem M. AboRas

**Affiliations:** Department of Electrical Power and Machines, Faculty of Engineering, Alexandria University, Alexandria, 21544 Egypt

**Keywords:** Power Loss, Distributed Generation, Metaheuristic Optimization, PVDG, Dingo Optimization Algorithm (DOA), Hybrid Renewable Energy Systems, Smart Dispatch, Energy science and technology, Engineering

## Abstract

The paper develops a comprehensive model for optimal allocation and sizing of DG units using the Dingo Optimization Algorithm (DOA) targeting active power loss reduction with a concomitant improvement in voltage stability across both distribution (DN) and transmission networks (TN). The novel methodology evaluates the effectiveness of operation of renewable DG units under two different network structures. In the IEEE 33-node DN, DG units comprising PV are optimally placed, with consideration for a constant amount of reactive compensation, considering the practical limitations of the used inverters. In the IEEE 118-node TN, which is a more complicated, meshed grid, PV alone, as well as hybrid configurations combining both PV and Wind technologies, operating at an optimal power factor of 0.95 are considered. In addition, a dispatch factor of 0.3 is applied for hybrid systems under light load to mitigate the high penetration of the renewable energy DG units. Results from the evaluation of IEEE 33-bus network indicate the effectiveness of DOA in securing substantial reductions in active power losses at percentages of 81.63%, 48.37%, and 79.45% in cases of normal, light, and heavy loadings, respectively, coupled with marked improvement in the voltage stability level of the power grid. For instance, during regular operations, the lowest Voltage Stability Index (VSI) rose from a critical point of 0.695 to a safe point of 0.898, whereas the highest Voltage Deviation Index (VDI) fell from 0.087 to 0.027. Application of the algorithm to the 118-bus TN indicates that placing three PV DG units optimally results in reductions in active power losses by 16.98%, 8.39%, and 16.24% in normal, light, and heavy loadings, respectively. Incorporating a hybrid system involving three PV units and three wind power DG units reduces the active power losses to 18.07% in the case of normal loading and 23.98% for heavy loading. Finally, deployment of the dispatch factor achieves a positive reduction in power losses of 12.61% in the light load scenario. Additionally, network stability improved in the hybrid topology when the network was under high traffic loads, leading to the minimum Voltage Deviation Index (VDI) being reduced from 0.1015 to 0.0863, while the maximum Fast Voltage Stability Index (FVSI) was 0.2817.

## Introduction

The power system has a big influence on how a nation can be safer and more capable. The power grid is the nation’s most important and complex asset. It includes generation, transmission (TN), and distribution (DN) systems, and they are often separated from each other following the relaxation rule^1,2^. Addressing the distribution and the transmission systems, they are the most vulnerable sector of the power grid, dealing with voltage failure and stability issues^3^. Engineers and researchers are putting efforts and time to resolve these issues and find effective and applicable solutions. Nowadays, the implementation of Distributed Generation (DG) is expanding due to its efficiency and applicability. DGs are separated into four types, Type 1, specifically Photovoltaic Distributed Generation (PVDG) is widely adapted worldwide due to the demand of using green energy and reducing the greenhouse gases (GHG)^4^. PVDG injects active power in the network, helping the voltage profile of the whole system and relieving stress on the network lines and helps stabilize and enhance power quality respecting the boundaries and limitations^5,6^. However, the selection of the locations and sizes of these DGs are very crucial in meeting these objectives; wrong allocation or an error in the power capability can cause a failure and put the network in a dangerous, unstable environment. To avoid this, engineers and researchers have studied, experimented and invested in using metaheuristic algorithms to tackle these allocations problems^7^.

### Literature review

Many researchers have studied the use of PVDGs and how to optimize it to increase the system quality and performance. Ref^8^. utilized the Archimedes optimization algorithm (AOA) to calculate and find the optimal locations and sizing of PVs in distribution systems. The research focused on reducing greenhouse gas emissions by using loss sensitivity parameters and AOA to get the optimal size and location for the PVs. Albadi et al.^9,10^ applied particle swarm optimization to get the optimal sizing and location for their PV in distribution network. The goal was to reduce transmission line loss and enhancing the voltage profile working on the network of Masirah Island in Oman as their case study. Moving toward the hybrid systems, Ali et al.^11^ investigated the integration of Wind Turbine and PVs utilizing the white shark optimization (WSO) on three distribution network (DN) systems, IEEE 33, 69, and 85-bus. The approach succeeded in reducing power losses and costs, respecting the system limitations. Khenissi et al.^12^ also examined sizing and allocation of PVDGs on a modified IEEE 14-bus network using Particle Swarm Optimization (PSO) and Genetic Algorithm (GA) to reduce the overall power loss and optimize voltage profile, accounting for load and environment disturbances. Other studies, like Ali et al.^13^ utilized loss sensitivity parameters and the ant lion optimization algorithm (ALOA) to optimize DG placement in a 69-bus radial distribution network. Also Ahmed et al.^14^ examined single and multiple PVDGs integration using augmented grey wolf algorithm on IEEE 69 bus system, showing promising results on loss reduction. Similarly, Almutairi et al.^15^ maximized the performance of distribution networks by optimally allocating PV-green distributed generation. Ahmadi et al.^16^ focused locally on using solar panel on rooftops, working the GA to minimize losses and reduce the country’s reliance on imported power. Khasanov et al.^17^ introduced the artificial ecosystem-based optimization opposition-based learning (AEO-OBL) algorithm. It was tested on IEEE 33 and 85-bus systems; it avoided the local optima dilemma and reduced total power losses better than other algorithms. Purlu and Belgin^18^ put the GA and PSO into comparison with the aim of zero carbon emissions by the year 2050. Their work on IEEE 33-bus distribution network showed that DGs operating at optimal power factors performed better than operating with unity PF, with PSO showing better search quality and convergence speed than the GA. Tukkee et al.^19^ utilized the firefly algorithm (FA) and fast voltage stability index (FVSI) for the integration of solar PV. Testing on the IEEE 33-bus DN, FA achieved loss reduction of 6.46%, slightly better than GA’s 6%. Addressing of the complexity of the Fourth Industry Revolution, Mubarak et al.^20^ used FA-PSO, a hybrid algorithm, to obtain optimal solutions for growth while adhering to technical boundaries and limitations. Khan et al.^21^ wrote about stability concerns using the honey badger algorithm (HBA). In comparison with WOA and GWO on networks like IEEE 33 and 69-bus, HBA had faster convergence, sometimes within two or three iterations, and successfully minimized power losses. Mahfoud et al.^22^ proposed the use of a hybrid more complex algorithm, the QODELFA, mixing Lévy flights with differential evolution. Working on radial systems like IEEE 33, 69, and 118-bus showed that the QODELFA reduced active power loss and improved the voltage stability better than existing traditional methods. Finally, Nowdeh et al.^23^ proposed a multi-objective hybrid teaching-learning-based optimization grey wolf optimizer (MOHTLBOGWO) for renewable implementation. The results showed that multi-objective optimization provides a more accurate network performance representation than single-objective methods. The strategy presented superior convergence speed, enhanced reliability and cost savings compared to other algorithms like GWO and TLBO.

### Research gap and contribution

This paper’s main contributions can be summed up as follows:

- An optimization framework that requires only one metaheuristic solver and can be applied to both radial distribution and meshed transmission networks.
- Renewable DG was distributed using the Dingo Optimization Algorithm, which performed well in large, severely constrained power systems.
- Considering light-load conditions and methods for halting reverse power flow, which are not frequently discussed in current DG planning studies.
- The IEEE 33-bus and IEEE 118-bus test systems with various loading conditions were used to determine the optimal distribution strategy for PV and hybrid PV-wind DGs.
- A thorough performance analysis that considers how well it lowers power loss, enhances voltage profiles, and improves voltage stability.

**Table 1:** Comparative analysis of DG allocation studies.

Feature	Typical DN-Focused Studies	Typical TN-Focused Studies	Proposed DOA-Based Framework
Network Type	Radial DN only	Meshed TN only	**DN + TN in one framework**
Test Systems	IEEE 33/69	IEEE 14/118	**IEEE 33 + IEEE 118**
DG Types	PV only	Conventional or PV only	**PV + Hybrid PV–Wind**
Optimization Solver	GA/PSO/GWO	NR-based heuristics	**Single DOA framework**
Light-Load Analysis	Rarely considered	Mostly ignored	**Explicitly analyzed**

A significant limitation of traditional metaheuristic algorithms is their lack of topological scalability. As empirically demonstrated in Section "Comparative performance analysis (selection of solver)", conventional solvers (GA, PSO, and GWO) exhibit inferior convergence and higher residual losses compared to the DOA even on the simple IEEE 33-bus radial network. This initial performance gap strongly indicates these algorithms would prematurely converge in the meshed IEEE 118-bus transmission network, where the solution space is orders of magnitude larger and contains complex cyclic loops.

Conversely, the Dingo Optimization Algorithm (DOA) overcomes these challenges by dynamically balancing local attacking and global encircling. Managed by the survival parameter *b*, this mathematical structure enables the DOA to bypass local optima, delivering a unified, robust solver capable of scaling seamlessly across both DN and TN topologies.

## Problem formulation

### The objective function

The primary goal of introducing DGs in distribution and transmission networks is reducing the active power losses. This objective function shows the total active power loss across all the network’s branches and is calculated mathematically using Equation (1)

1 $$\begin{aligned} OF_{min} = \sum _{i}^{nb} |I_{i}|^2 R_{i} \end{aligned}$$

where $R_{i}$ = ith line resistance, *n* *b* = overall network lines, and $|I_i|$ = ith line curent

While Equation (1) concentrates exclusively on minimizing active power (P) loss, the overall voltage stability of both distribution and transmission networks is improved. In the physics of power systems, voltage stability and power loss are highly correlated; a considerable reduction in the line currents is needed to minimize the branch losses $I^2R$ demands. This is supported analytically: for a branch with impedance $Z = R + jX$, the voltage drop is approximated as $\Delta V \approx \frac{PR + QX}{V}$, establishing a direct mathematical link between power loss minimization and voltage deviation^6,12^. Consequently, the voltage drop across the network impedances is diminished, which helps enhance the stability margin of the system (which can be observed in the improvement of the Voltage Stability Index) and flatten the voltage profile (which can be observed in the improvement of the Voltage Deviation Index). In addition, instead of using a multi-objective approach, this methodology ensures stability of the grid voltage by imposing strict voltage limits (detailed in Section “Voltage limitations”). Any violation of these safe, strongly imposed boundaries are penalized by the DOA, guaranteeing a final result that minimizes the power losses while securely operating within an improved stability margin

### Equality constraints

The optimization problem is subject to power flow balance equations, which ensure that the power supplied equals the load demand plus losses. To address the distinct topological characteristics of the networks under study, two different power flow methods are employed:

- For the IEEE 33-bus Distribution Network: Due to its radial structure and high R/X ratio, the Backward/Forward Sweep (BFS) method is utilized. This iterative method relies on Kirchhoff’s Voltage Law (KVL) and Kirchhoff’s Current Law (KCL) to update branch currents and bus voltages efficiently without forming a Jacobian matrix.
- For the IEEE 118-bus Transmission System: Due to its meshed nature and complexity, the Newton-Raphson (NR) method is employed. The active (P) and reactive (Q) power match equations used in this method are described in Equations (2) and (3).

2 $$\begin{aligned} P_{Gi} = P_{Di} + \sum _{j=1}^{nb} |V_i| |V_j| [G_{ij} \cos \theta _{ij} + B_{ij} \sin \theta _{ij}] \end{aligned}$$

3 $$\begin{aligned} Q_{Gi} = Q_{Di} + \sum _{j=1}^{nb} |V_i| |V_j| [G_{ij} \sin \theta _{ij} - B_{ij} \cos \theta _{ij}] \end{aligned}$$

where $P_{Gi}$ and $P_{Di}$ = active power generated and demanded,respectively, $V_{i}$ and $V_{j}$ = bus voltage values, $Q_{Gi}$ and $Q_{Di}$= reactive power generated and demanded, respectively, and $G_{ij}$ and $B_{ij}$ = line conductance and susceptance, respectively.

### Inequality constraints

These constraints ensure the system operates safely and securely. Strict voltage boundaries must be applied to all buses, along with restrictions concerning the PVDG units

To numerically impose these constraints in the system’s logic, a constant penalty coefficient is used. For any dingo solution that violates the voltage or operational ratings, a heavy penalty is integrated into its fitness evaluation, encouraging the DOA to discard it and confine the search only within the secure, restricted operating region.

#### Voltage limitations

Equation (4) sets precise voltage boundaries, representing the upper and lower limits within which the voltage levels should operate.

4 $$\begin{aligned} V_{max} \ge V_i \ge V_{min} \end{aligned}$$

where $V_{max}$ and $V_{min}$ = maximum and minimum voltage values, respectively.

#### Photovoltaic distributed generation limits

The optimal size of PVDG units requires compliance to sizing boundaries, specified by the upper and lower limits, as shown in Equation (5).

5 $$\begin{aligned} PVDG_{max} \ge PVDG_i \ge PVDG_{min} \end{aligned}$$

where $PVDG_{max}$ and $PVDG_{min}$ = maximum and minimum active power injection value, respectively.

### Voltage deviation index (VDI)

The Voltage Deviation Index (VDI) is used to quantify the quality of the system. The VDI calculates the deviation of the voltage across all buses from the standard value of 1.0 p.u. The lower the VDI, the flatter and more stable voltage profile is. The VDI is mathematically calculated by summing the squared differences between the nominal voltage and the actual voltage at each bus across the network using Equation (6):

6 $$\begin{aligned} VDI = \sum _{i=1}^{n_b} (V_{ref} - V_i)^2 \end{aligned}$$

where $V_{ref} = 1.0$ p.u. is the nominal reference voltage, $V_i$ is the voltage magnitude at bus *i*, and $n_b$ is the total number of buses.

### Voltage stability index (VSI)

Voltage stability is an important term of power system safety, especially under heavy loading conditions. The Voltage Stability Index (VSI) is used to identify the weakest buses and most exposed to voltage failure. A VSI value near 1 represents a stable line, but a value leaning towards 0 shows a line nearing collapse. In this research, the VSI is monitored to make sure that the integration of hybrid DGs increases the system’s stability margin. For the radial distribution network, this is evaluated using the Chakravorty VSI for a branch connecting sending bus *s* to receiving bus *r* as shown in Equation(7):

7 $$\begin{aligned} VSI(r) = |V_s|^4 - 4(P_r X - Q_r R)^2 - 4(P_r R + Q_r X)|V_s|^2 \end{aligned}$$

where $|V_s|$ is the voltage magnitude at the sending bus, *R* and *X* are the branch resistance and reactance, and $P_r$ and $Q_r$ are the active and reactive power at the receiving bus.

To evaluate the voltage stability of the Transmission network, the Fast Voltage Stability Index (FVSI) is utilized as the VSI metric, perfectly suited for complex, meshed transmission network in this study. The FVSI for a transmission line connecting bus *i* to bus *j* is mathematically defined as shown in Equation(8):

8 $$\begin{aligned} FVSI_{ij} = \frac{4Z^2 Q_j}{V_i^2 X} \end{aligned}$$

where *Z* is the line impedance, *X* is the line reactance, $Q_j$ is the reactive power at the receiving bus *j*, and $V_i$ is the voltage magnitude at the sending bus *i*.

## Dingo Optimization Algorithm

The Dingo Optimization Algorithm (DOA) gets the inspiration from the hunting techniques and clever social dynamics of dingoes^24^. Packs of 10 to 15 members, dingoes follow the most dominant individual, the alpha, while lower ranking like betas submit to this hierarchy. Scouts play an important role by reconnoitering the surroundings and alert the others if there’s a threat. Their special hunting technique consists of well coordinated actions like pursuing, pestering, surrounding, and attacking prey. The DOA utilize these aspects to optimize resource placement, copying the coordination of the pack. By integrating dingo social coordination, the algorithm shows effectiveness and adaptability in solving optimization tasks, adding a novel dimension to the field.

### Mathematical modeling of the Dingo Optimization Algorithm

Imitating the circling, hunting, attacking, and searching behaviors of dingoes during the optimization^25^, the DOA applies a mathematical model that mimics the adaptive techniques dingoes use in search of prey^25,26^. Implementing these natural behaviors, the DOA showed performance improvements and effectiveness in tackling the optimization problems across multiple contexts.

#### Encircling:

The encircling behavior, involving the strategic positioning and coordination of the pack members, is modeled using mathematical Equations (9)–(13).

9 $$\begin{aligned} \vec {D}_C = \left| \vec {A} \cdot \vec {P}_p(x) - \vec {P}(x) \right| \end{aligned}$$

10 $$\begin{aligned} \vec {P}(i+1) = \vec {P}_p(i) - \vec {B} \cdot \vec {D}(d) \end{aligned}$$

11 $$\begin{aligned} \vec {A} = 2 \cdot \vec {a}_1 \end{aligned}$$

12 $$\begin{aligned} \vec {B} = 2\vec {b} \cdot \vec {a}_2 - \vec {b} \end{aligned}$$

13 $$\begin{aligned} \vec {b} = 3 - \left( I \cdot \left( \frac{3}{I_{max}} \right) \right) \end{aligned}$$

Here, $\vec {D}_{d}$ represent the separation between the prey and dingo, $\vec {P}_{P}$ and $\vec {P}$ represent prey and dingo position vectors, respectively, $\vec {A}$ and $\vec {B}$ are coefficient vectors where $\vec {a}_{1}$ and $\vec {a}_{2}$ are random vector in [0, 1],The variable $\vec {b}$ decreases linearly from 3 to 0, and *I* is the current iteration up to $I_{max}$. These equations represents the unified encircling of prey, stimulated the effectiveness of the behavior of dingoes within the optimization structure^27^.

#### Hunting:

In optimization, the position of the prey is generally unknown to each of the dingoes. However, DOA supposes the pack has some knowledge of possible locations, embedding this in the hunting technique. The Alpha dingo ($\alpha$) sets the hunting pace, assisted by other dingoes like the Beta ($\beta$). Formally, the algorithm sets a reference based on the position of the best two search dingoes, with the rest of the pack recalibrating their positions based on Equations (14)–(19).

14 $$\begin{aligned} \vec {D}_{\alpha } = \left| \vec {A}_1 \cdot \vec {P}_{\alpha } - \vec {P} \right| \end{aligned}$$

15 $$\begin{aligned} \vec {D}_{\beta } = \left| \vec {A}_2 \cdot \vec {P}_{\beta } - \vec {P} \right| \end{aligned}$$

16 $$\begin{aligned} \vec {D}_{\textrm{o}} = \left| \vec {A}_3 \cdot \vec {P}_{\textrm{o}} - \vec {P} \right| \end{aligned}$$

17 $$\begin{aligned} \vec {P}_1 = \left| \vec {P}_{\alpha } - \vec {B} \cdot \vec {D}_{\alpha } \right| \end{aligned}$$

18 $$\begin{aligned} \vec {P}_2 = \left| \vec {P}_{\beta } - \vec {B} \cdot \vec {D}_{\beta } \right| \end{aligned}$$

19 $$\begin{aligned} \vec {P}_3 = \left| \vec {P}_{\textrm{o}} - \vec {B} \cdot \vec {D}_{\textrm{o}} \right| \end{aligned}$$

These equations integrate the core of the DOA’s hunting techniques, showing how pack members update their movement and location based on the Alpha and collective information. Modeling this collective wisdom, the algorithm effectively improves the search procedure. The intensity coefficient for each individual dingo is calculated by using Equations (20)–(22).

20 $$\begin{aligned} \vec {I}_{\alpha } = \log \left( \frac{1}{F_{\alpha } - (1E-100)} + 1 \right) \end{aligned}$$

21 $$\begin{aligned} \vec {I}_{\beta } = \log \left( \frac{1}{F_{\beta } - (1E-100)} + 1 \right) \end{aligned}$$

22 $$\begin{aligned} \vec {I}_{\textrm{o}} = \log \left( \frac{1}{F_{\textrm{o}} - (1E-100)} + 1 \right) \end{aligned}$$

where $F_{\alpha }$ and $F_{\beta }$ = $\alpha$ and $\beta$ dingo fitness values, respectively, and $F_{o}$ = other dingoes fitness value. These equations provided the mathematical calculations for assessing dingo intensities within the DOA.

#### Attacking prey:

After the hunting, if no position update is required, the dingo attacks. Mathematically, the variable *b* decreases in linearity from 3 to 0, simultaneously reducing the intervals of the random variable $D_\alpha$ between $[-3b, 3b]$. When *b* decreases, the capacity movement scope reduces. When $D_\alpha$ is in $[-1, 1]$, the dingo goes to a position between its current position and the prey’s location. This technique represents the dingo’s resolution, balancing between exploration and exploitation to efficiently find an optimal solution.

#### Searching:

Dingoes use the pack’s cooperative position to locate, track, and attack the prey. The random variable represented by $\vec {B}$ regulates this dynamic in the DOA: values less than −1 show that the prey is diverging. in contrast, a value bigger than 1 shows the pack is approaching, activating the global scanning routine. Also, the coefficient $\vec {A}$ promotes exploration by creating a random integer between 0 and 3, as shown in Equation (11), to support variable prey weights. This predictive framework simplifies the analysis of the distance gap described in Equation (10).

### Application of the Dingo Optimization Algorithm for optimal DG allocation

- Initialize the population of dingoes with randomized locations and velocities within the specified search space.
- Evaluate the fitness of each dingo using the objective function focused on network performance parameters (e.g., reduction of power loss and voltage stability) resulting from DG placement
- Flag the dingo with the best solutions as a global best
- Recalibrate the position and velocity of each dingo agent based on its knowledge and the global best solution.
- Apply limitations bounds to make sure that the recalibrated placements of the dingo agents remain within the valid region.
- Calculate the fitness for each individual dingo.
- Compare the new fitness to the previous values to modify the personal best solutions for each dingo.
- If a new personal fitness is found to be better than the global, update it as the new global best.
- Repeat steps 4–8 until the convergence threshold are met.
- Select the best dingo as the final solution and place the DGs to the system based on this results.

**Fig. 1:**
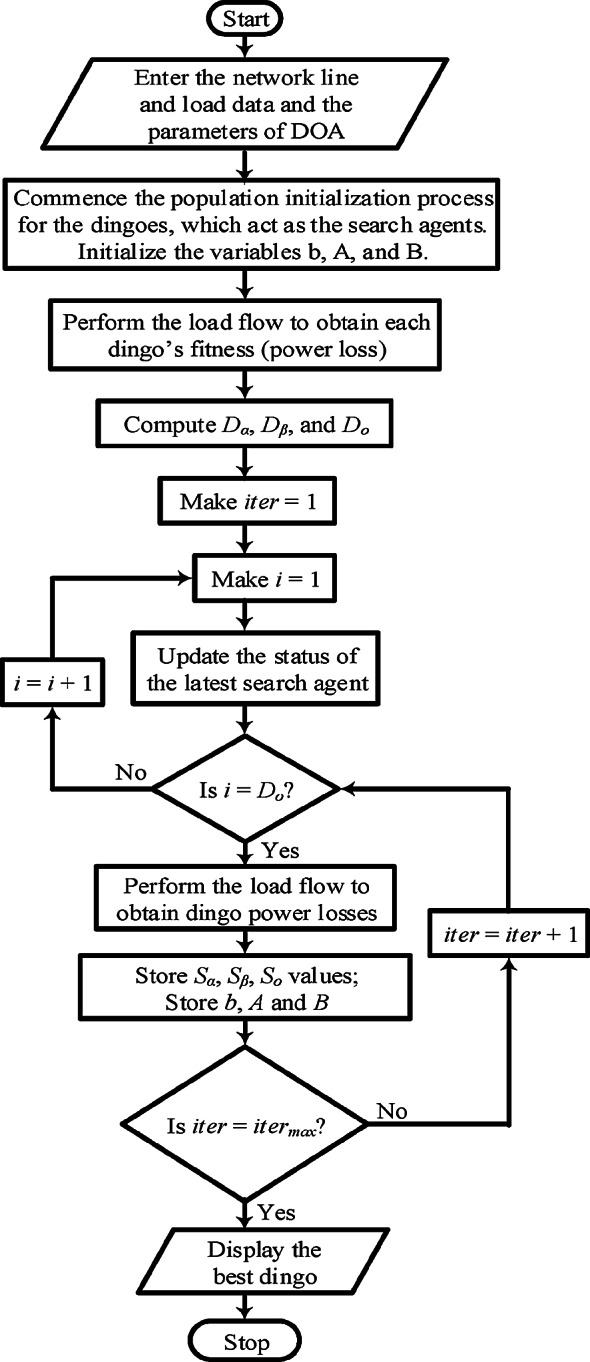
Implementation of the DOA for PVDG placement.

Figure 1 shows a flowchart explaining the procedure. For the placement of PVDGs in the IEEE 33-bus radial distribution network and IEEE 118-bus meshed transmission network utilizing the DOA, specific parameters were appointed, including the following:

- Dingoes population: A population size of approximately 800 individuals was set as the work force of the system.
- Number of iterations: The DOA was given 200 iterations to find optimal solutions.
- Crossover Rate: To efficiently tackle the competing objectives between local exploitation and global exploration, a crossover rate of 0.7 is set.
- Mutation Rate: to ensure system diversity between its population, a mutation rate value of 0.1 was set
- Pressure selection : to control the probability of selecting superior dingoes, the pressure selection was hold at a value of 2.
- Convergence Criteria: The optimization process was considered complete upon the improvement of the objective function is reduced below a set threshold, often determined at 1%.

These parameters were precisely set and implemented in the DOA architecture to optimize the placement of these PVDGs in the IEEE 33-bus distribution network (DN) and the IEEE 118-bus transmission network (TN).

## Results and discussion

The optimization framework was done in the MATLAB environment. To know the most effective and efficient algorithm to work with, a thorough comparison analysis was done between four metaheuristic optimization techniques, Genetic Algorithm (GA), Grey Wolf Optimizer (GWO), Particle Swarm Optimization (PSO) and Dingo Optimization Algorithm (DOA). On the basis of the results of this comparative analysis, DOA was chosen as the solver due to its superior searching mechanism and consistent results. The DOA is chosen for the full covered analysis on both the IEEE 33-bus radial distribution network and the IEEE 118-bus meshed transmission system.

### Comparative performance analysis (selection of solver)

To validate that the DOA is the right choice for our research, two distinct tests were simulated on the standard IEEE 33-bus system under normal loading conditions: an Efficiency Test and a Robustness Test.

To ensure true and unbiased comparative comparison, a unified initialization procedure was used for all metaheuristic algorithms. Using a synchronized initial population matrix, this method alignment ensures that the comparison is fairly justified. This ensures the superiority of the proposed DOA is entirely due to its advanced social hierarchy logic and the balance between global search and local attacking and hunting mechanisms instead of a lucky initial guess

#### Efficiency test

The first test shows us the convergence speed and solution quality of the algorithms with very limited computational power (20 Agents, 50 Iterations).

**Fig. 2:**
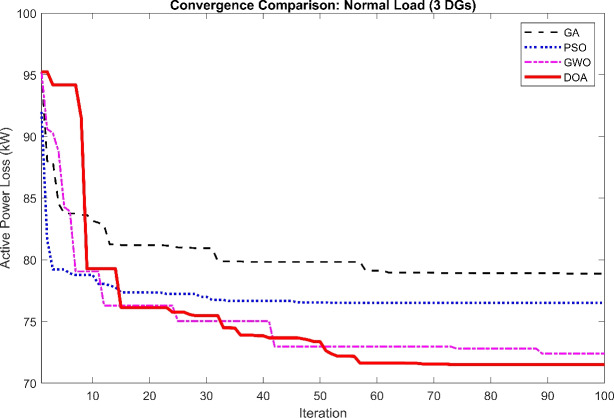
Convergence curve comparison between four optimizations algorithms.

**Fig. 3:**
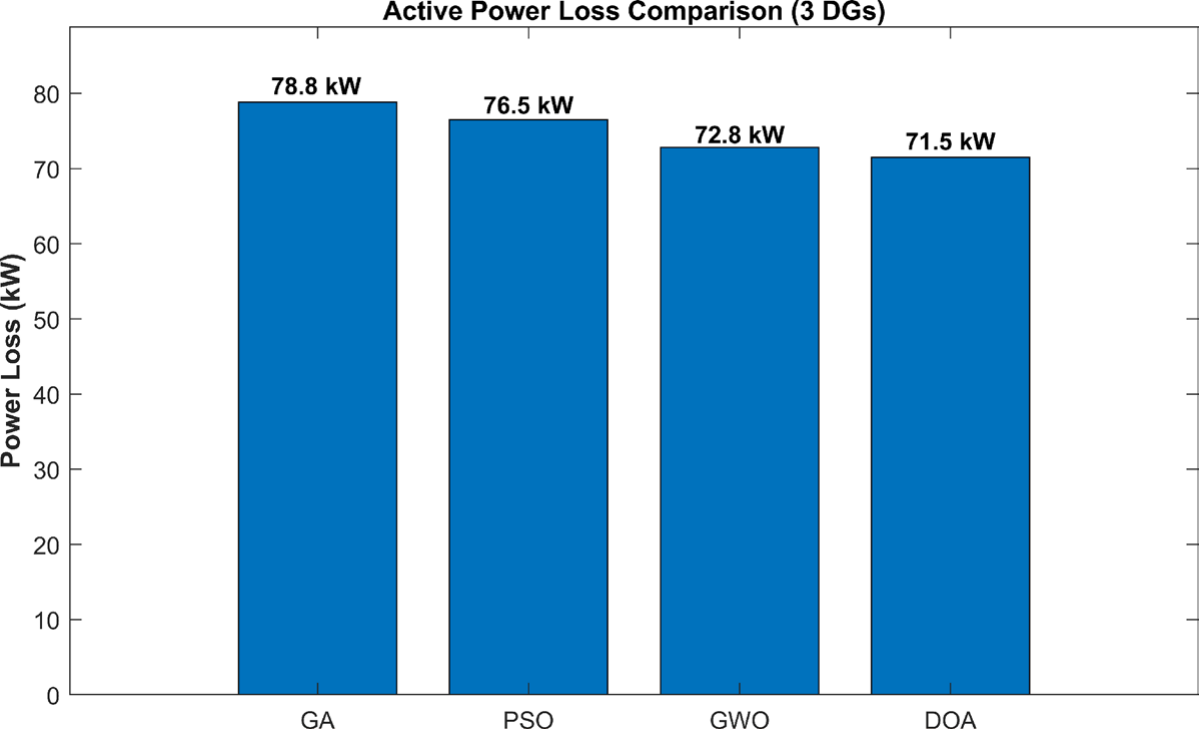
Active power loss in efficiencytest.

**Fig. 4:**
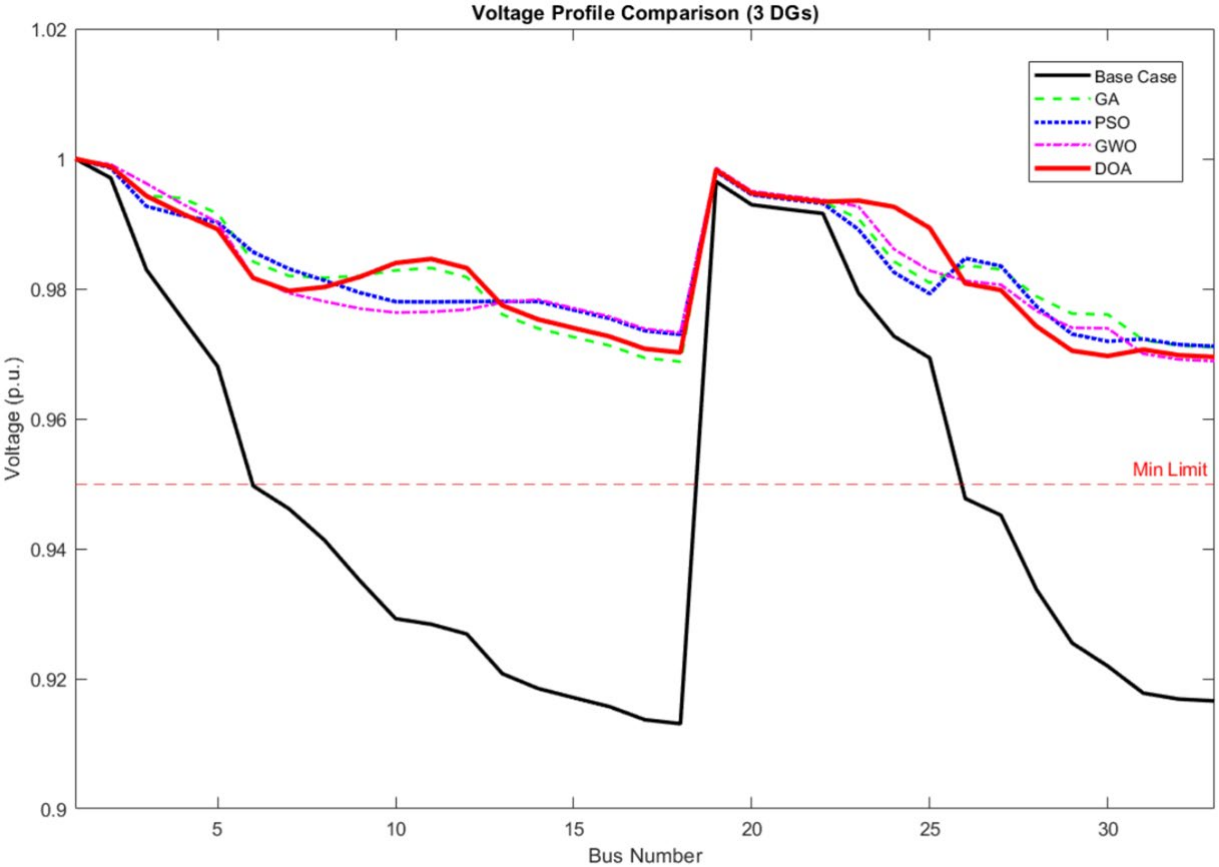
Voltage profile comparison between four optimization algorithms.

- Convergence Characteristics The convergence curve of the four algorithms is shown in Fig. 2. Due to the synchronized initial values, every algorithm starts the optimization procedures from the same starting active power loss value around 95.1 kW. This strategy demonstrates a clear visual representation of the exploitation efficiency and search speed of the DOA in comparison to standard algorithms. As seen, the DOA demonstrates higher convergence characteristics compared to GA, PSO, and GWO. While the other algorithms face early sluggishness or slower descent, the DOA rapidly navigates the search space and stabilizes at a lower objective function value (active power loss) earlier in the process. This proves a strong balance between exploration and exploitation, allowing it to avoid the local optimal solutions that trapped the GA and PSO algorithms.
- Active Power Loss Comparison Fig. 3 presents the minimum active power losses explored by each algorithm. The DOA achieved the minimum power loss of 71.5 kW, better than GWO (72.8 kW), PSO (76.5 kW), and GA (78.8 kW). This is equivalent to a 64.6% reduction in losses for the DOA, confirming its efficiency in locating high quality solutions even with a limited population size and iterations
- Voltage Profile Improvement The importance of the optimal placement on the network voltage profile is shown in Fig. 4. The base case (black line) faces significant voltage drops specially in bus 18 and towards the end of the radial feeder. The DOA solution (red line) remarkably elevates the voltage magnitude across all buses, holding a profile closer to the reference 1.0 p.u. in comparison to the other optimization algorithms GA, PSO, and GWO.

**Table 2:** Active power losses comparison between algorithms.

Algorithm	Power Losses (KW)
GA	78.8
PSO	76.5
GWO	72.8
DOA	71.5

#### Robustness test

To correctly judge the stability and reliability of these algorithms, a Robustness Test was done with higher parameters (50 Agents, 100 Iterations), averaged over 30 independent runs.

**Fig. 5:**
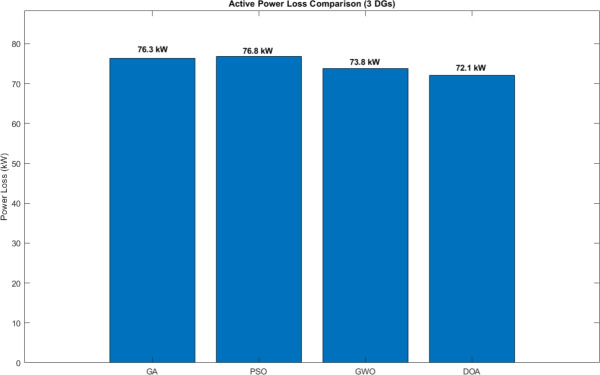
Active power loss comparison in the robustness test.

- Average Active Power Loss The average active power losses we got from the robustness test are shown in Fig. 5. The DOA got the lowest average power loss of 72.1 kW as a 64.3% reduction percentage, surpassing GWO (73.8 kW), PSO (76.8 kW), and GA (76.3 kW). The power of the DOA to continue finding a minimal power losses with a larger population validates its scalability.

**Table 3:** Active power losses comparison between algorithms.

Algorithm	Power Losses (KW)
GA	76.3
PSO	76.8
GWO	73.8
DOA	72.1

### IEEE 33-bus system results

The DOA is used in the IEEE 33-bus radial distribution network, using the Backward/Forward Sweep (BFS) power flow method to get the best solution for PVDG integration. The optimization process identified the most strategic buses for DG placement, putting in mind the nodes that are most vulnerable to voltage drops and power losses. To enhance voltage stability more and to have a practical real world scenario, the optimization merged a fixed reactive power injection of 250 kVar at each PVDG location as a representation of the inverter power. This reactive support proved to be effective in improving the power factor at buses in need; specifically, it resulted in improved power factors of 0.95 at Bus 14, 0.97 at Bus 24, and 0.97 at Bus 30 which are all in the safe margin.

#### Optimal sizing and placement

The optimization process showed remarkable consistency in selecting the 3 buses where the PV will be integrated within the network. When the Dingo Optimization Algorithm (DOA) was set to run independently for each of the three loading conditions (Normal, Light, and Heavy), it consistently selected Buses 14, 24, and 30 as the optimal locations for DG integration. This consistency confirms that these specific buses are the most vulnerable points in the radial feeder and the most in need of these 3 PVDGs, regardless of the load level.

To calculate a single, robust size for practical implementation that will be effective across all scenarios, the algorithm utilized a Weighted Sum approach. The weights used in Equation (23) have been determined using the probability density function of the LDC for an average year, as well as the sizing criteria as stated in the distribution planning literature^14,18^. In statistical terms, the network runs under nominal circumstances for most part of the year (probability weight of 0.6), but occasionally experiences either light loads during off-peak times or heavy loads during peak periods (probability weight of 0.2 for each).

23 $$\begin{aligned} P_{DG}^{Final} = 0.2 \times P_{DG}^{Light} + 0.6 \times P_{DG}^{Normal} + 0.2 \times P_{DG}^{Heavy} \end{aligned}$$

For optimal sizing purposes, considering the nominal state (0.6) for the final DG size can be considered a major insurance policy in order to avoid over-sizing. If equal weight was given to the heavy load state, the result would be PV size that leads to huge overvoltage issues and unfavorable power flow reversals when dealing with the 20% of the time where the grid is underloaded. Hence, 0.2/0.6/0.2 ratio helps in optimizing active power loss and ensures voltage stability at all levels. The specific optimization results for each loading condition, which helps as inputs for this calculation, are detailed below:

- Normal Load: The optimal sizes that the DOA got were 1057.87 kW at Bus 30, 1055.80 kW at Bus 24, and 755.70 kW at Bus 14.
- Light Load: The optimal sizes which are obviously smaller due to low demand scenario were 375.06 kW at Bus 14, 535.57 kW at Bus 24, and 522.75 kW at Bus 30.
- Heavy Load: The optimal sizes under huge system stress were found to be 830.80 kW (Bus 14), 1182.15 kW (Bus 30), and 1212.49 kW (Bus 24).

Having the inputs to the Weighted Sum equation, the final optimal sizes were calculated as 695 kW, 983 kW and 976 kW at Buses 14, 24, and 30, respectively. This accurate sizing make sure that enough active power injection to supply load system demand at designated buses, and relieve the grid’s stress while avoiding reverse power flow violations in light load scenario.

**Table 4:** Optimal sizes and location for the 3PVDGs in IEEE 33-bus system.

Bus	Power (kW)
14	695
24	983
30	976

#### Scenario 1: normal load voltage analysis

**Fig. 6:**
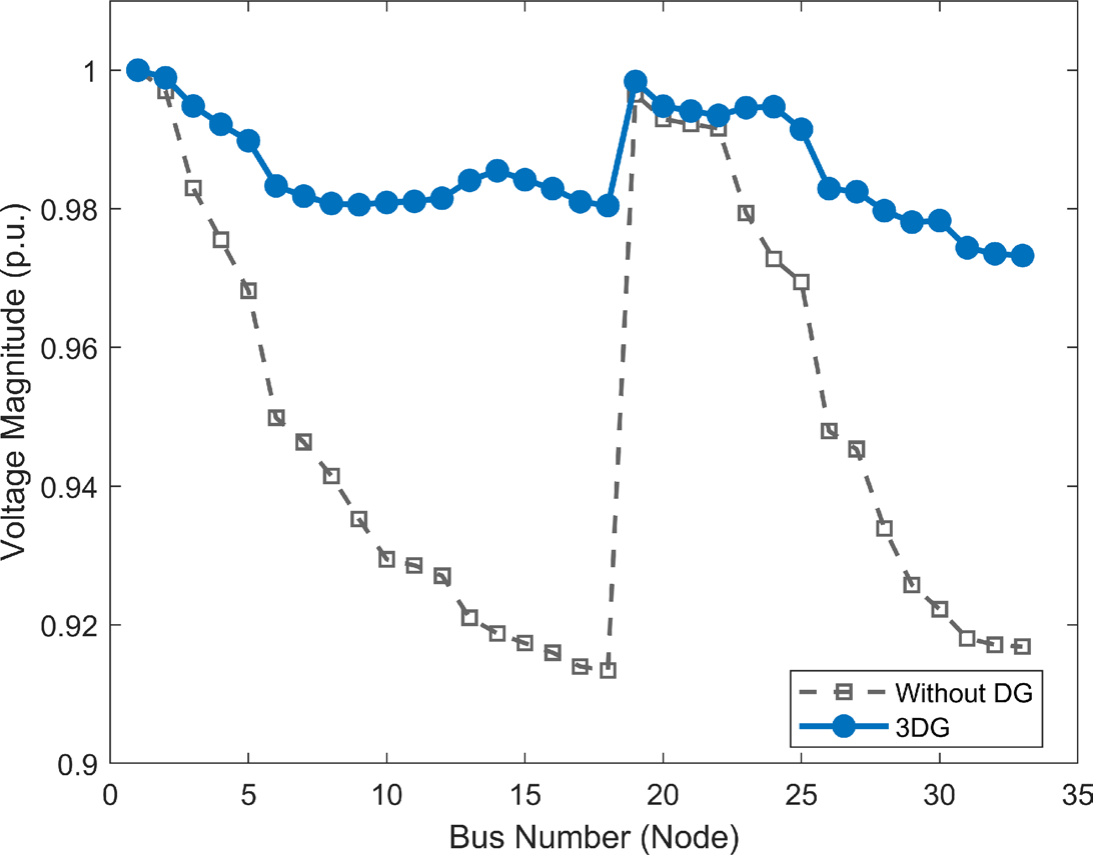
Voltage profile comparison under normal load condition.

**Fig. 7:**
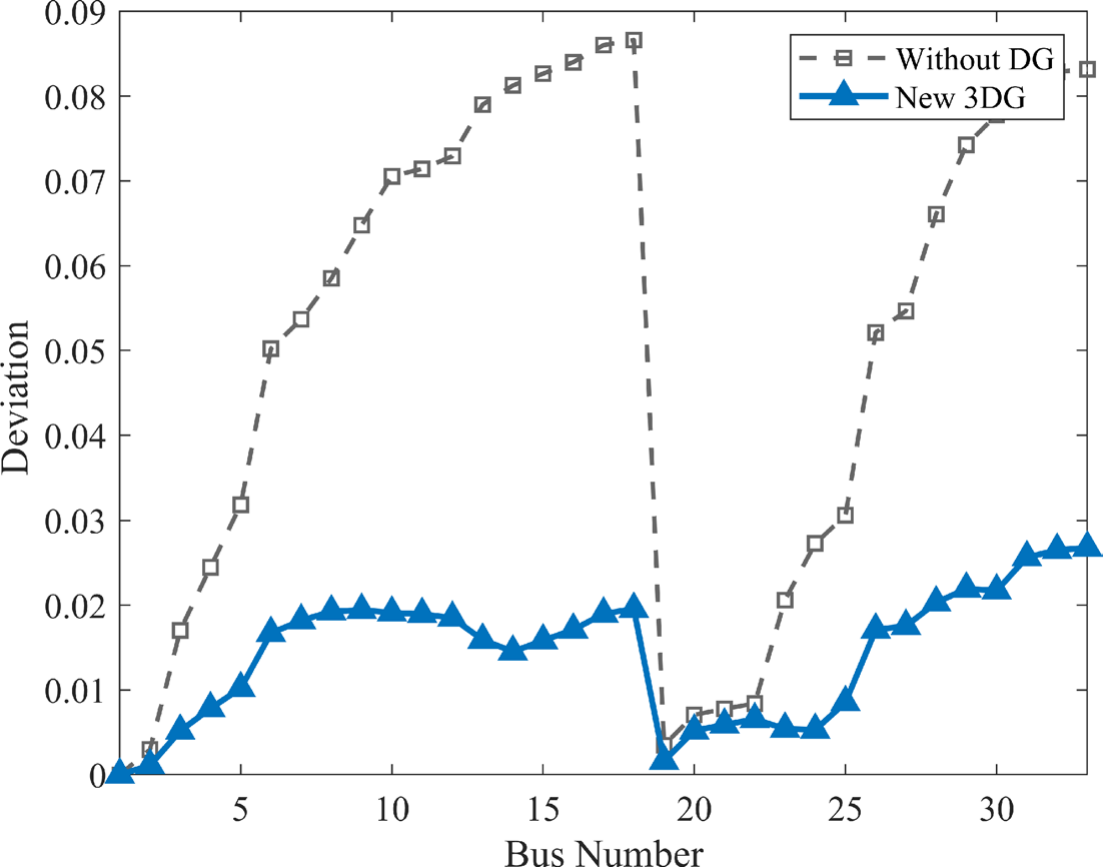
Voltage deviation index comparison under normal load condition.

**Fig. 8:**
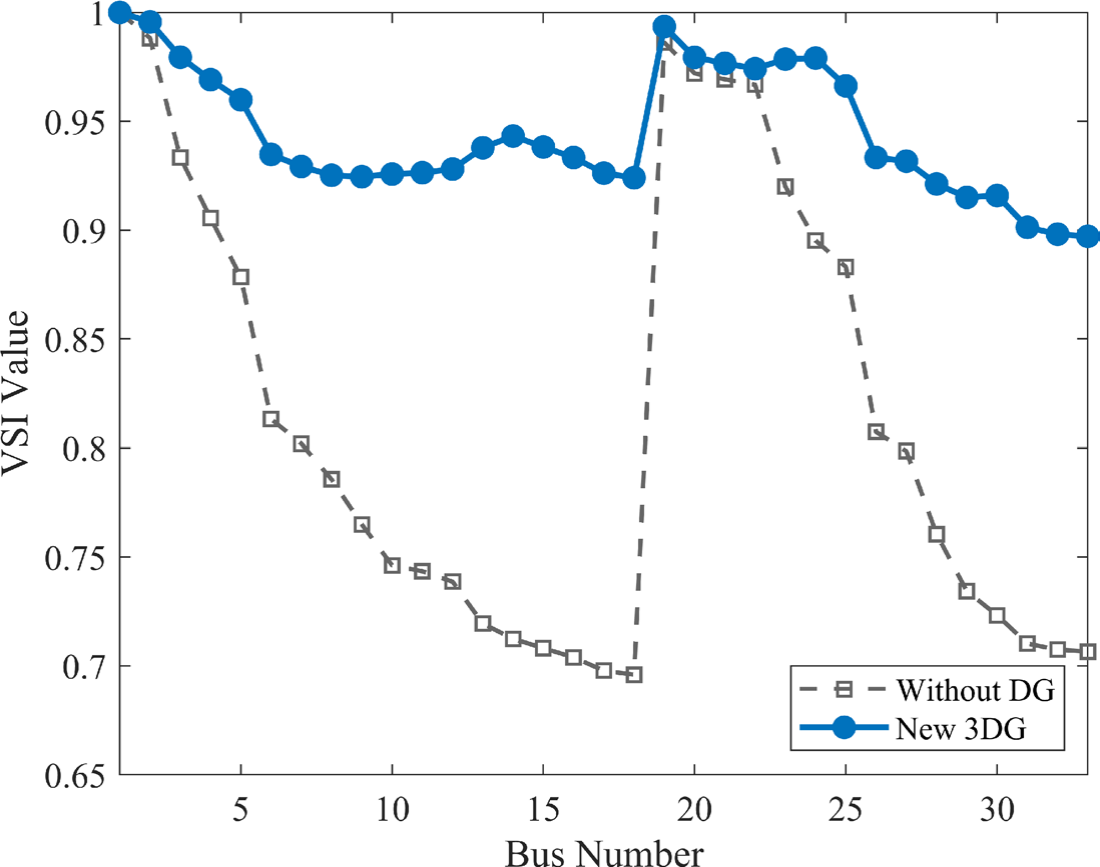
Voltage stability index comparison under normal load condition.

The voltage profile, as shown in Fig. 6, illustrates the comparison between base case and the implementation of PVDGs optimised by the DOA. Before the implementation of PVDG units, the voltage profile results was lower than voltage limits, with buses at the end of the feeder showing big voltage drops. The lowest voltage magnitude is shown as 0.9134 p.u. in base case, well below the standard limit of 0.95 p.u.. This shows a subpar voltage profile caused by the uncompensated radial structure. Yet, after the strategic implementations of the three DOA optimized PVDG units, an impressive improvement was seen across the network. The minimum voltage magnitude was raised to 0.9732 p.u., avoiding all under-voltage violations. The optimized profile remains flat and stable as shown in both VDI and VSI (Fig. 7, 8), showing the work of the calculated allocation in supporting the network’s voltage levels and stability.

#### Scenario 2: light load voltage analysis

**Fig. 9:**
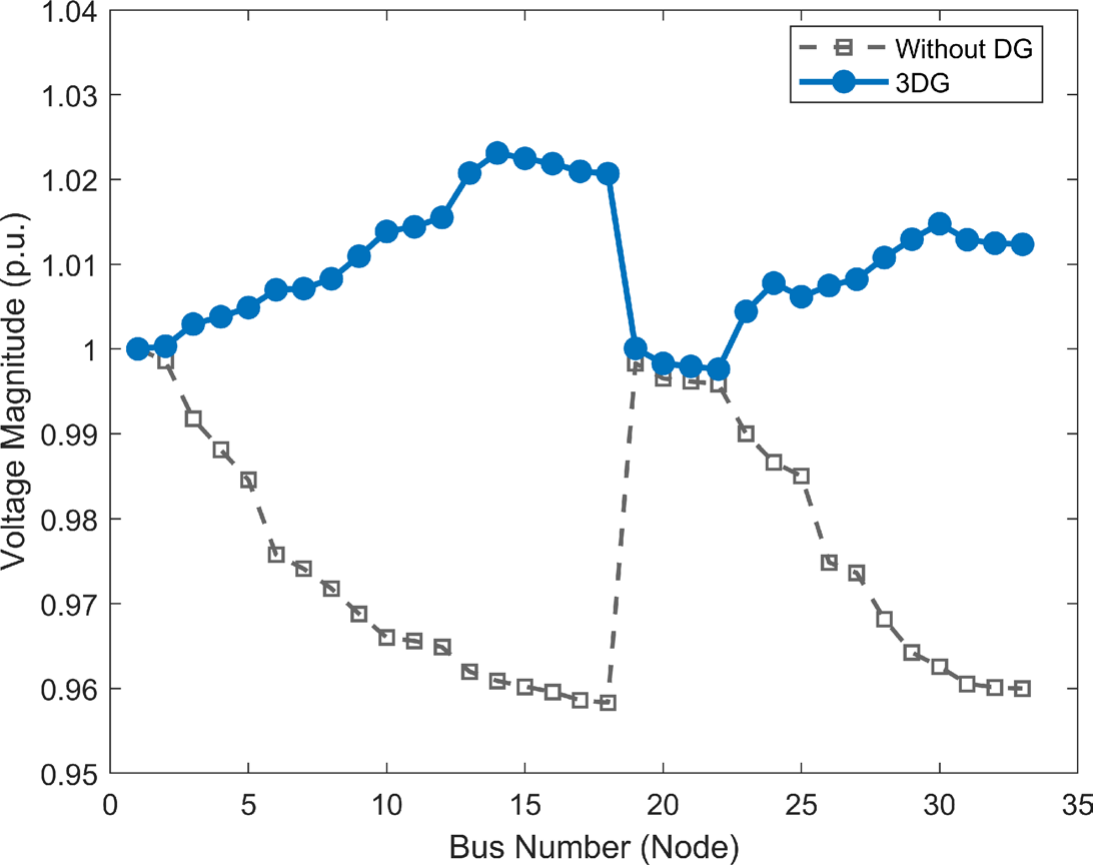
Voltage profile comparison under light load condition.

**Fig. 10:**
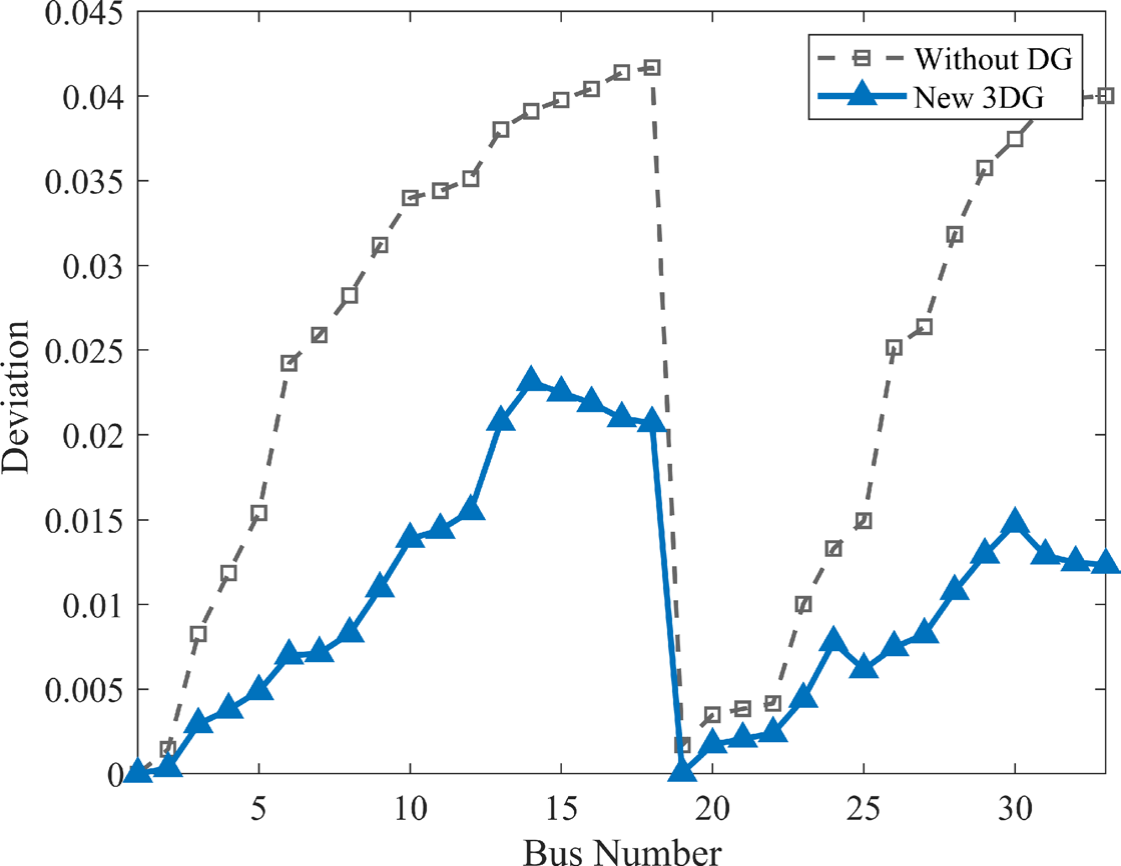
Voltage deviation index comparison under light load condition.

The research extended its study to the light load scenario (50% loading) to see the system’s behavior during off-peak periods. As shown in Fig. 9, the voltage profile faces a remarkable boost because of the PVDG deployment. While the VDI and the VSI as shown in Fig. 10, 11 shows good numbers indicating the stability and safety of the system In the base case, the lowest voltage was seen as 0.9583 p.u. While this is slightly within limits, the optimized allocation increased this minimum value to 0.9976 p.u.. This result is important because it shows the entire voltage profile nearly to the ideal reference of 1.0 p.u. up to 1.023 without exceeding the upper permissible limit of 1.05 p.u. This illustrates the flexibility of the DOA optimized PVDGs in improving the system performance without introducing over-voltage risks during low-demand periods.

**Fig. 11:**
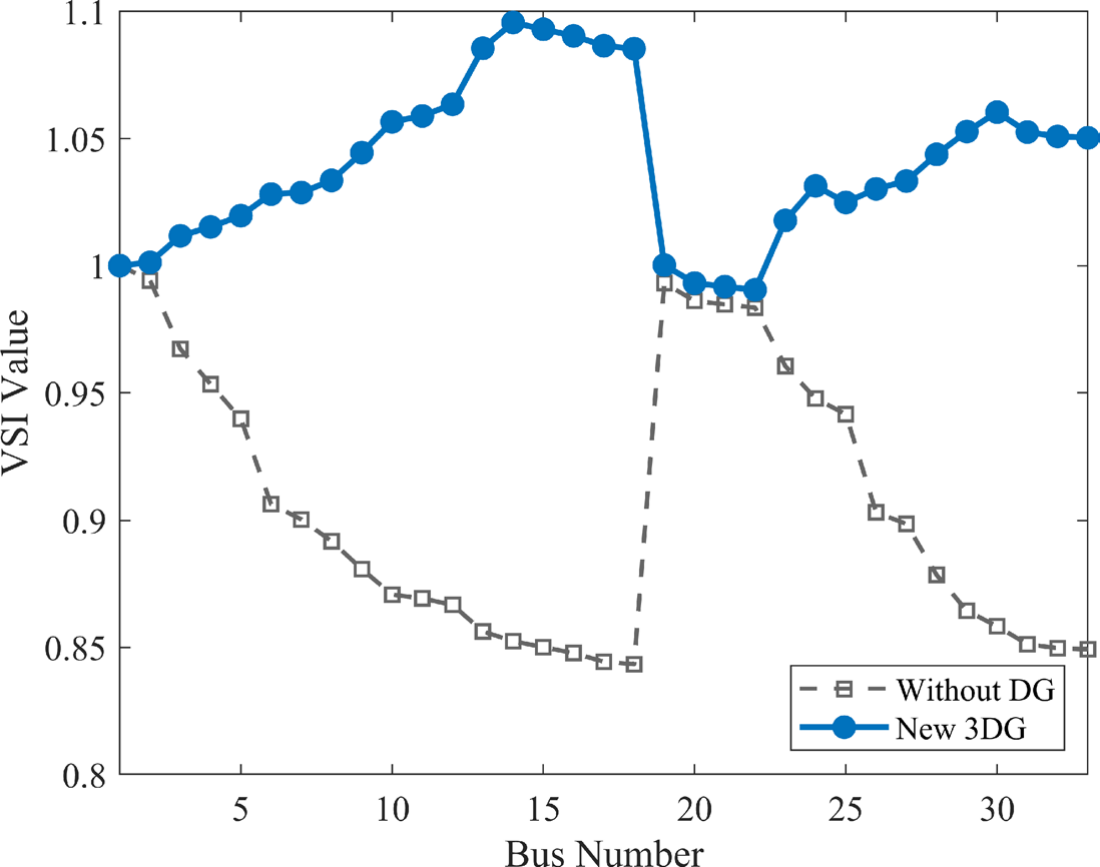
Voltage stability index comparison under light load condition.

#### Scenario 3: heavy load voltage analysis

**Fig. 12:**
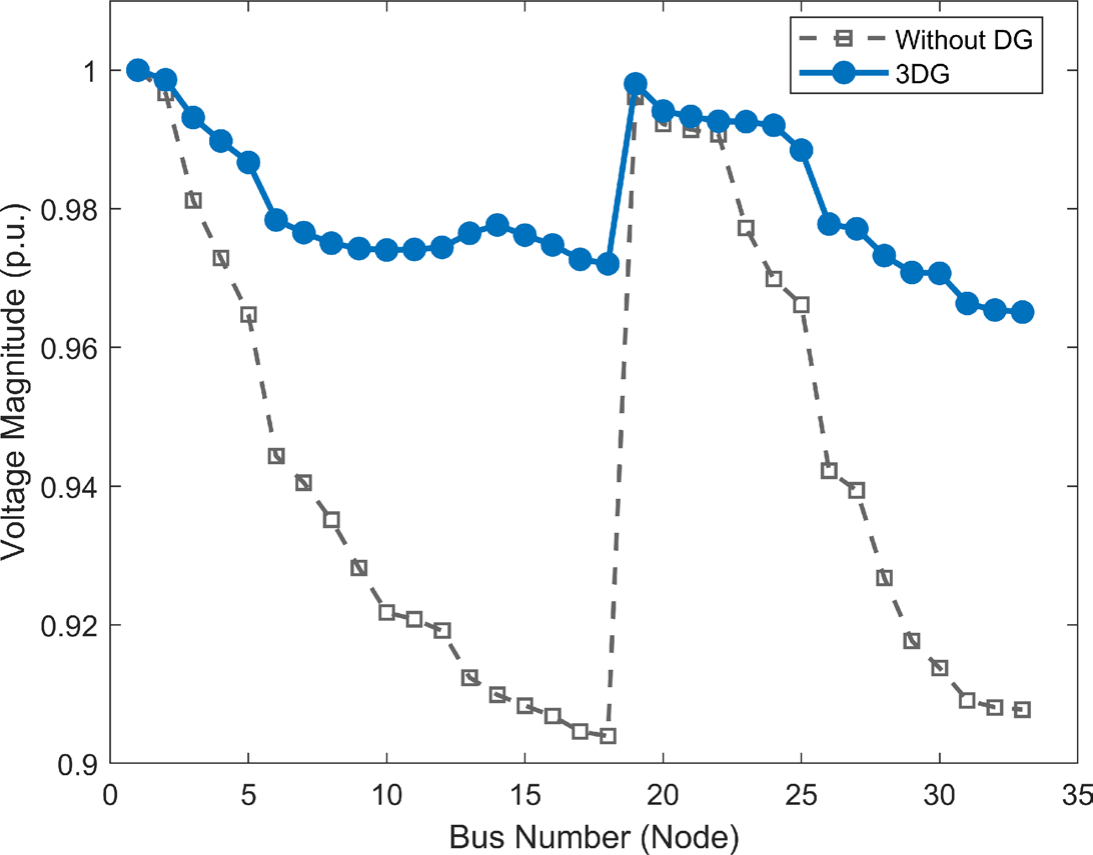
Voltage profile comparison under heavy load condition.

**Fig. 13:**
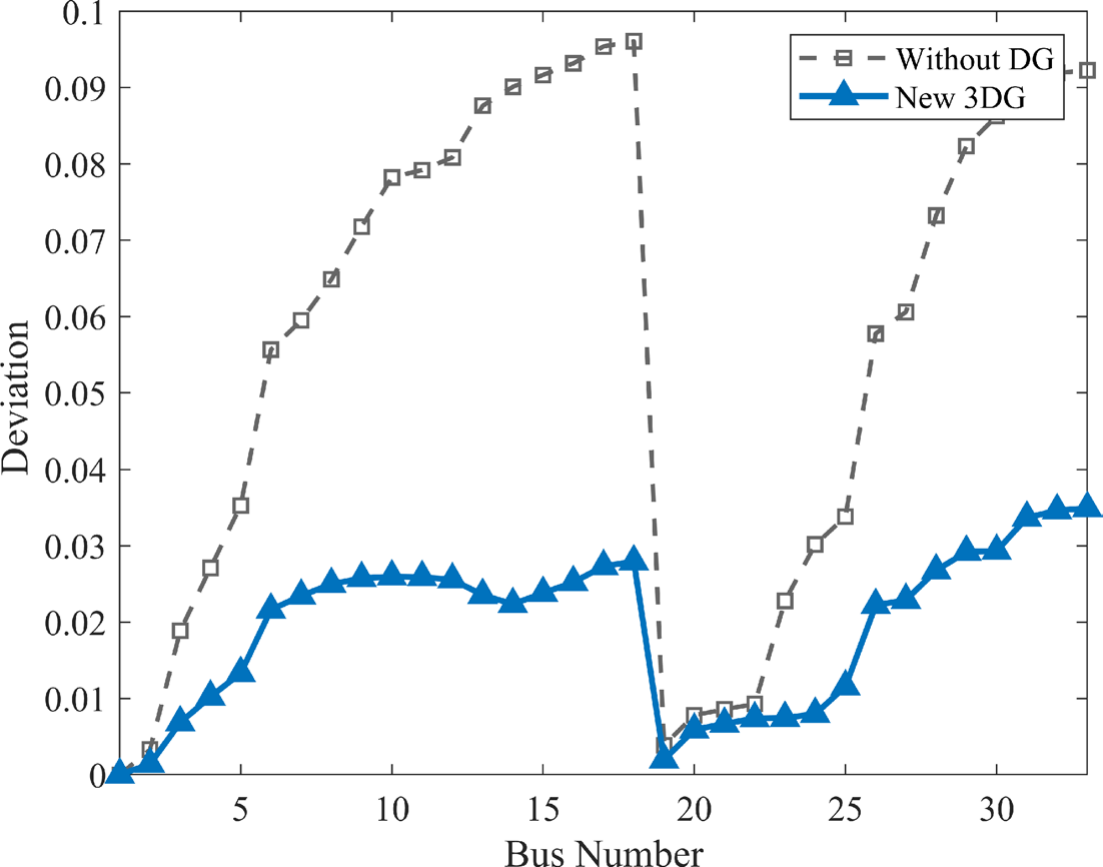
Voltage deviation index comparison under heavy load condition.

**Fig. 14:**
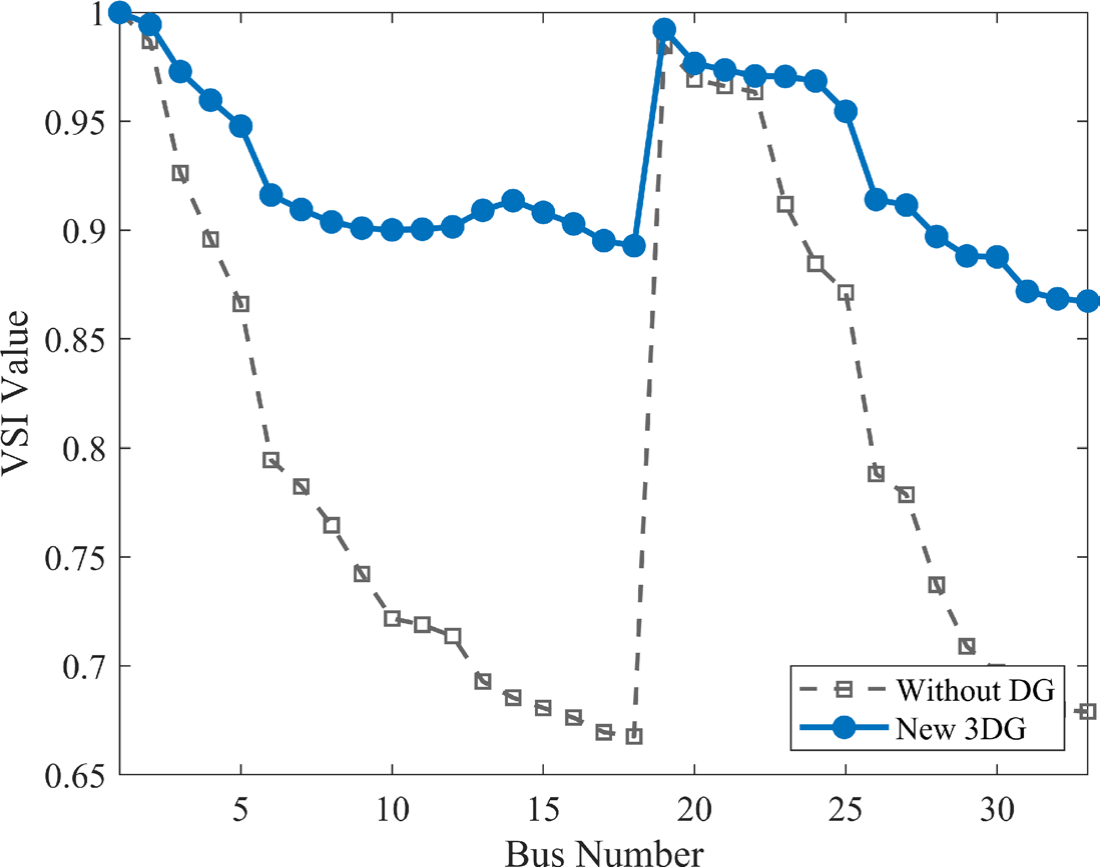
Voltage stability index comparison under heavy load condition.

With heavy load conditions (110% loading), the effects of the selected PVDGs became even more clear and visible. The base case system shows high voltage stress across the system buses, as shown in Fig. 12, with the lowest voltage value diving deep to a risky value of 0.9039 p.u., showing a high risk of failure due to instability. With the implementation of DOA optimized PVDGs, the lowest voltage magnitude was restored safely to 0.9651 p.u within the safe margins. This demonstrates a voltage lift of nearly 6.7%, which is a valuable margin for the distribution network under immense stress. By getting the damaged buses to a safe voltage value higher than 0.95 p.u, the addition of the PVDGs selected by the DOA helped tackling the issues and got the system to safely work within safe margins. These results highlight the capability of the DOA to maintain grid health as presented in VDI and VSI (Fig. 13, 14) under the most challenging operating conditions.

**Fig. 15:**
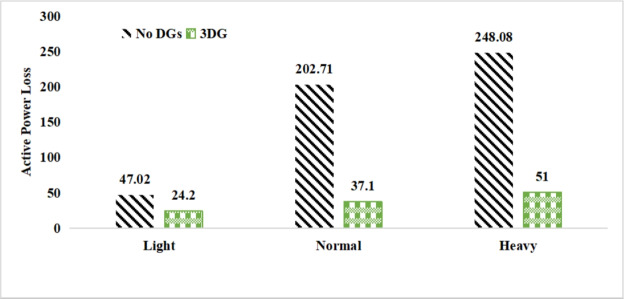
Total active power losses comparison under all loading conditions.

**Fig. 16:**
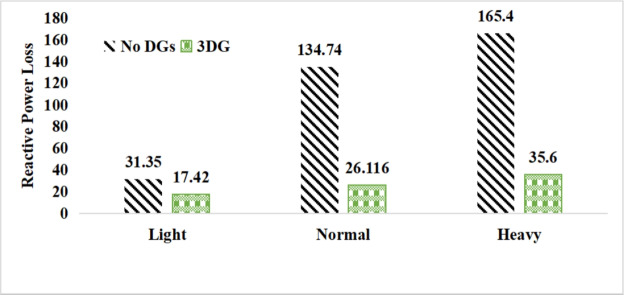
Total reactive power losses comparison under all loading conditions.

#### Active and reactive power loss analysis

The use of these PVDG units gave us magnificent improvements in network efficiency. This section shows the reduction in both active and reactive power losses, starting with total power losses and then getting to details with per branch power losses.

**Fig. 17:**
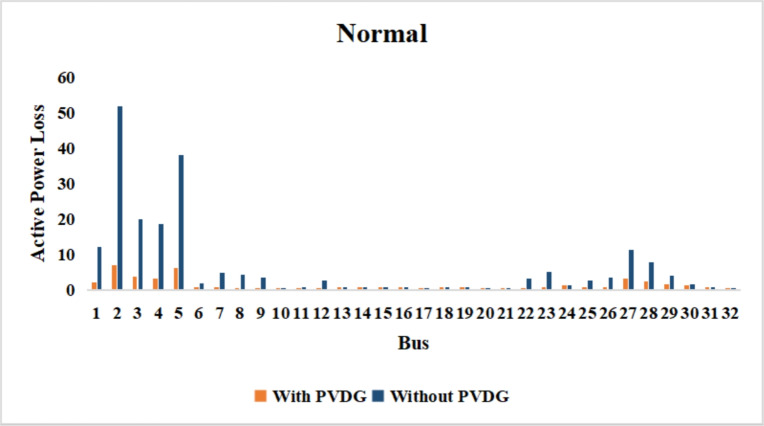
Active power losses across all branches in normal load condition.

**Fig. 18:**
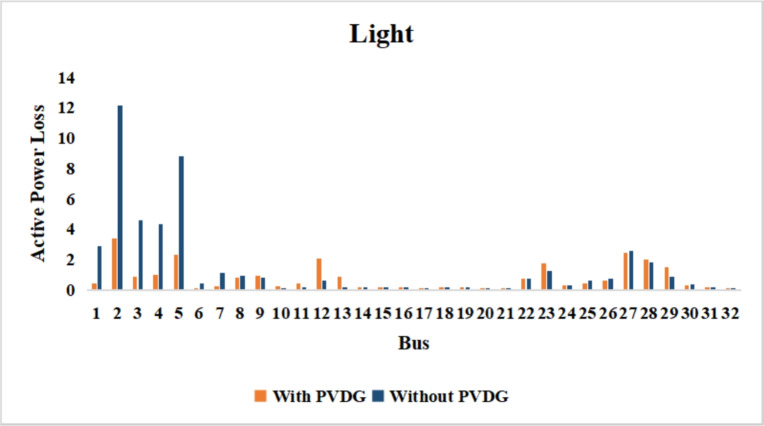
Active power losses across all branches in light load condition.

**Fig. 19:**
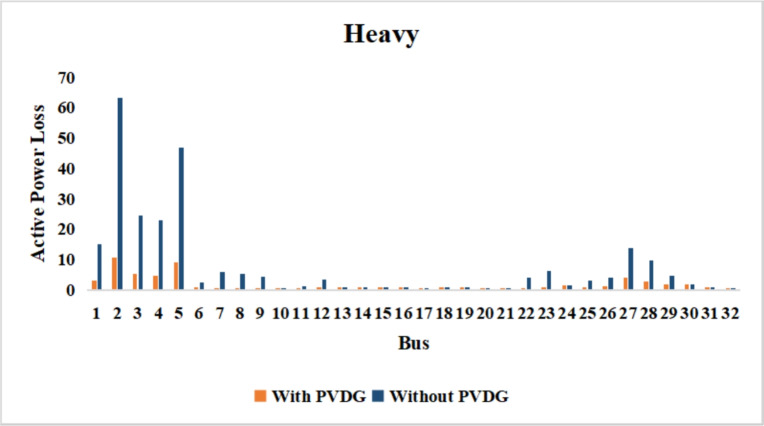
Active power losses across all branches in heavy load condition.

- Total Active and Reactive Power Loss Reduction Fig. 15 and Fig. 16 gives a good view of the total power losses across all three loading scenarios. In the Normal Load condition, the total active power loss was reduced from 202.71 kW to 37.1 kW, showing an 81.7% reduction percentage. Similar efficiency was seen under Heavy Load conditions, where losses dropped from 248.08 kW to 51.0 kW (79.43% reduction). Also with Light load, where the baseline losses were naturally lower (47.02 kW), the DOA optimization did a further reduction to 24.2 kW (48.5% reduction) The proposed method also significantly reduced the reactive power weight on the substation. As shown in Fig. 16, the total reactive loss under Heavy Load conditions decreased from 165.4 kVar to 35.6 kVar. This 78.5% reduction confirms the efficiency of the placement of PVDGs that helped minimizes the $I^2*X$ losses in the lines, contributing to the voltage stability improvements.
- Branch-wise Loss Minimization To see the impact of the three PVDGs implementation in the system, the losses were analyzed on a branch-by-branch basis. Fig. 17, 18 and 19 visualize the active power losses for each branch under the Normal, Light and Heavy Load scenario respectively. Before the compensation, the branches near the substation (specifically branches 1–2, 2–3, and 5–6) experienced the highest power losses resultant from the accumulation of current flow supplying downstream loads. For example, Branch 2 experienced high resistive losses in the base case. However, following the optimal injection of active power at Buses 14, 24, and 30, the current flow through these main feeder branches was reduced. As result, the active power loss in these branches is reduced significantly like shown in Fig. 17

**Fig. 20:**
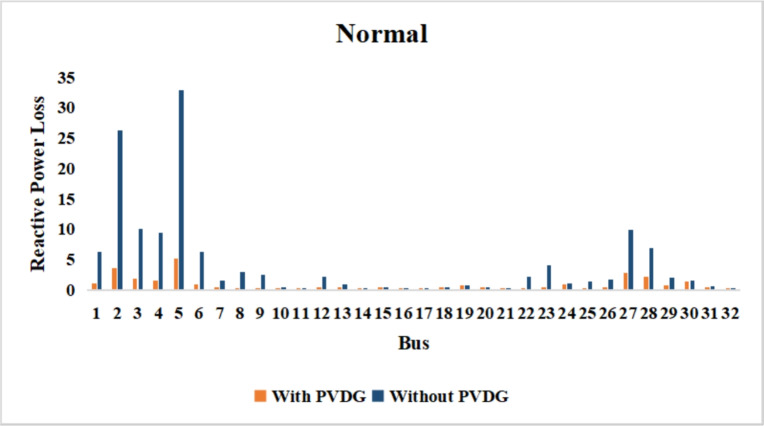
Reactive power losses across all branches in normal load condition.

**Fig. 21:**
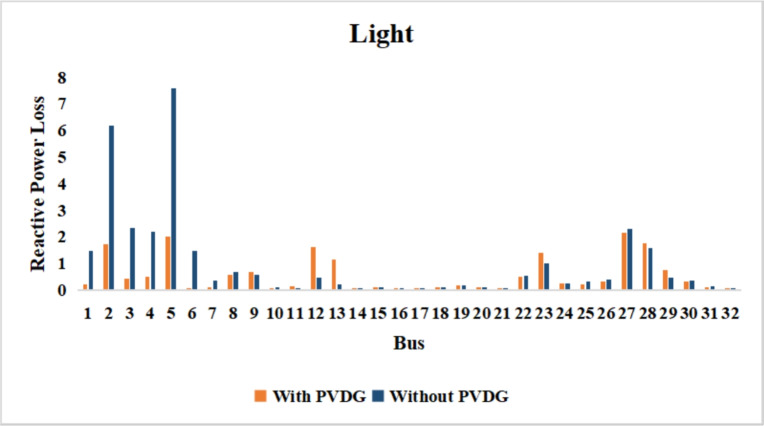
Reactive power losses across all branches in light load condition.

A similar results are seen for reactive power losses in Fig. 20, 21, and 22. The DGs are suppling a portion of the local power demand, relieving the stress on the first couple branches and reducing the reactive current flowing from the source and as a result minimizing the reactive drops across the network branches. This local relief mechanism is the most effective reason behind the global performance enhancement of the IEEE 33-bus system.

**Fig. 22:**
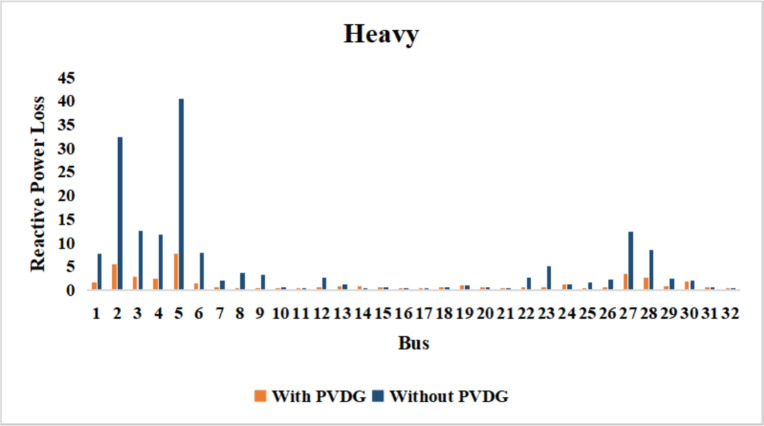
Reactive power losses across all branches in heavy load condition.

### IEEE 118-bus transmission system results (Scenario A: 3 PVDGs)

To see if we can expand our framework to bigger more complex systems, the DOA was put to test with the IEEE 118-bus transmission system. This massive meshed network represents a significant increase in complexity in comparison to the radial more simple distribution test case (IEEE 33-bus), this system components consist of 54 generators, 177 transmission lines, 9 transformers, and 91 loads with a total power demand of 4,242 MW^28^. To handle this complex topology, the Newton-Raphson (NR) power flow method was used not the Backward/Forward Sweep (BFS) algorithm that was used for the radial distribution system, the NR method is designed to solve the cyclic loops and multiple voltage controlled buses in meshed transmission networks scenarios where the BFS approach is mathematically ineffective. In this first scenario, the optimization algorithm run at a selected 1000 Dingo and 120 iterations to find the three perfect PVDGs locations and sizing.

#### Optimal sizing and placement

The optimization process successfully got the best solutions for PVDG placement in the meshed network. The optimal sizes for the transmission system were calculated by runnig the three loading scenarios and get their results and use them in the weighted sum formula. The objective function have the weighted active power losses across Light, Normal, and Heavy loading conditions ($W_{Light} = 0.2, W_{Normal}=0.6, W_{Heavy}=0.2$) in every iteration to ensure a balanced solution. As shown in Table 5, the DOA gave us three distinct buses for implementing the PVDGs. The algorithm got the optimal active power ratings that minimize the weighted loss while maintaining a safe constant Power Factor of 0.95 to provide necessary reactive power support. This way ensures that the calculated sizes are robust and applicable to all operating scenarios.

**Table 5:** Optimal sizes and location for the 3PVDGs in IEEE 118-bus system.

Bus	Power (MW)
36	73.06
40	112
53	92.50

#### Scenario 1: normal load voltage analysis

**Fig. 23:**
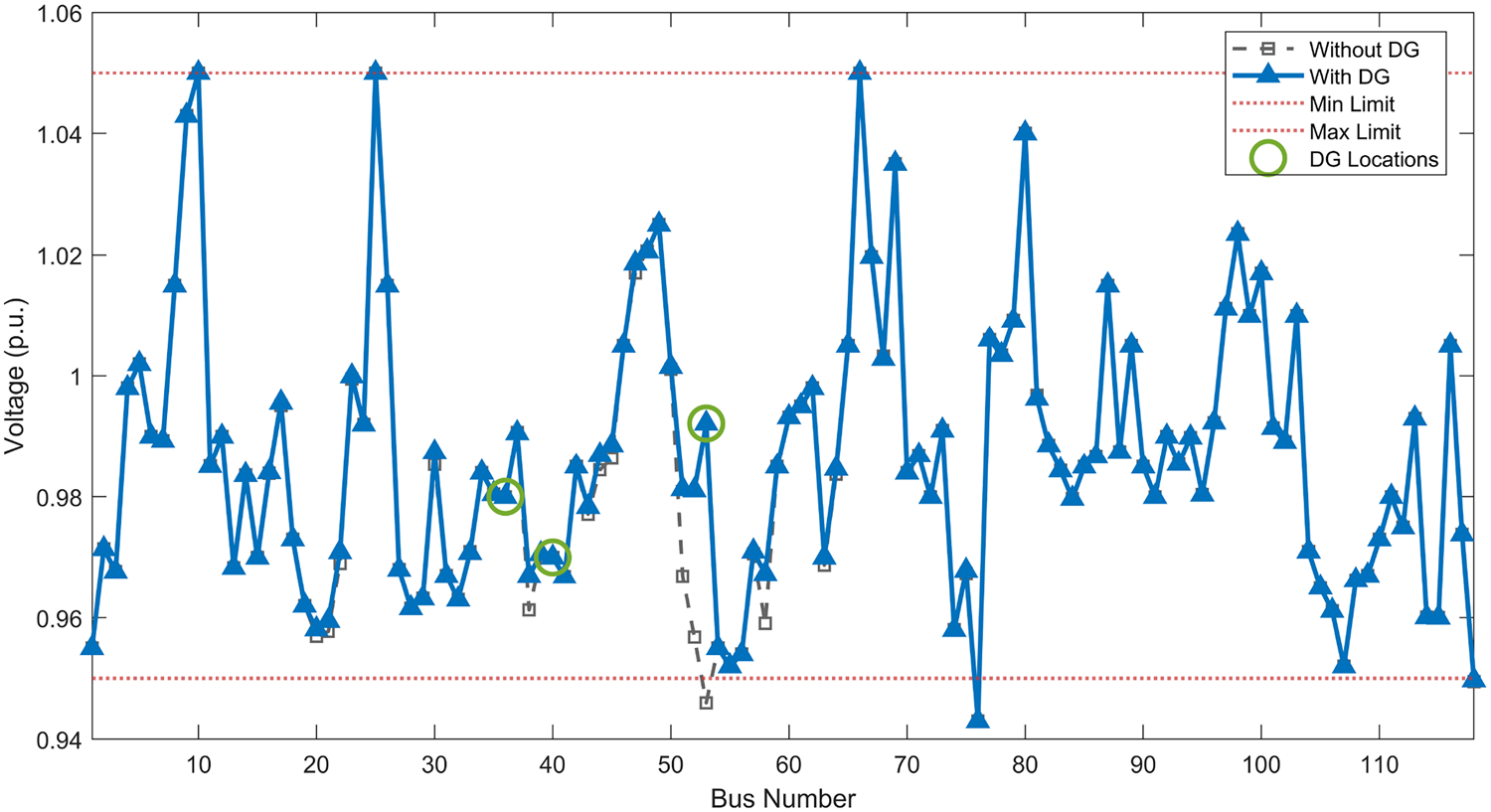
Voltage profile comparison under normal load condition.

**Fig. 24:**
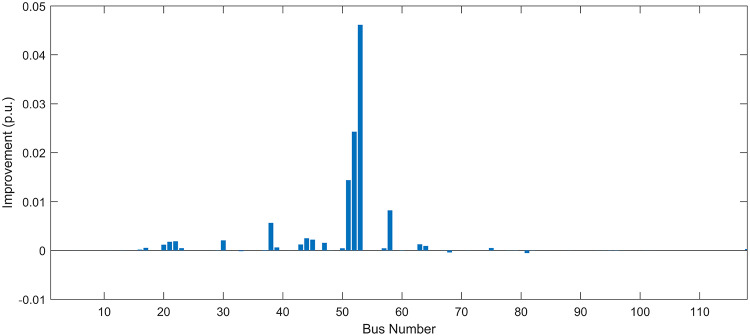
Voltage lift under normal load condition.

**Fig. 25:**
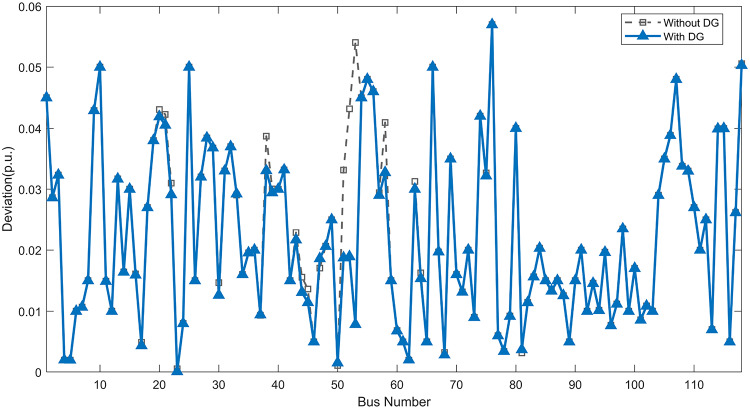
Voltage deviation index comparison under normal load condition.

**Fig. 26:**
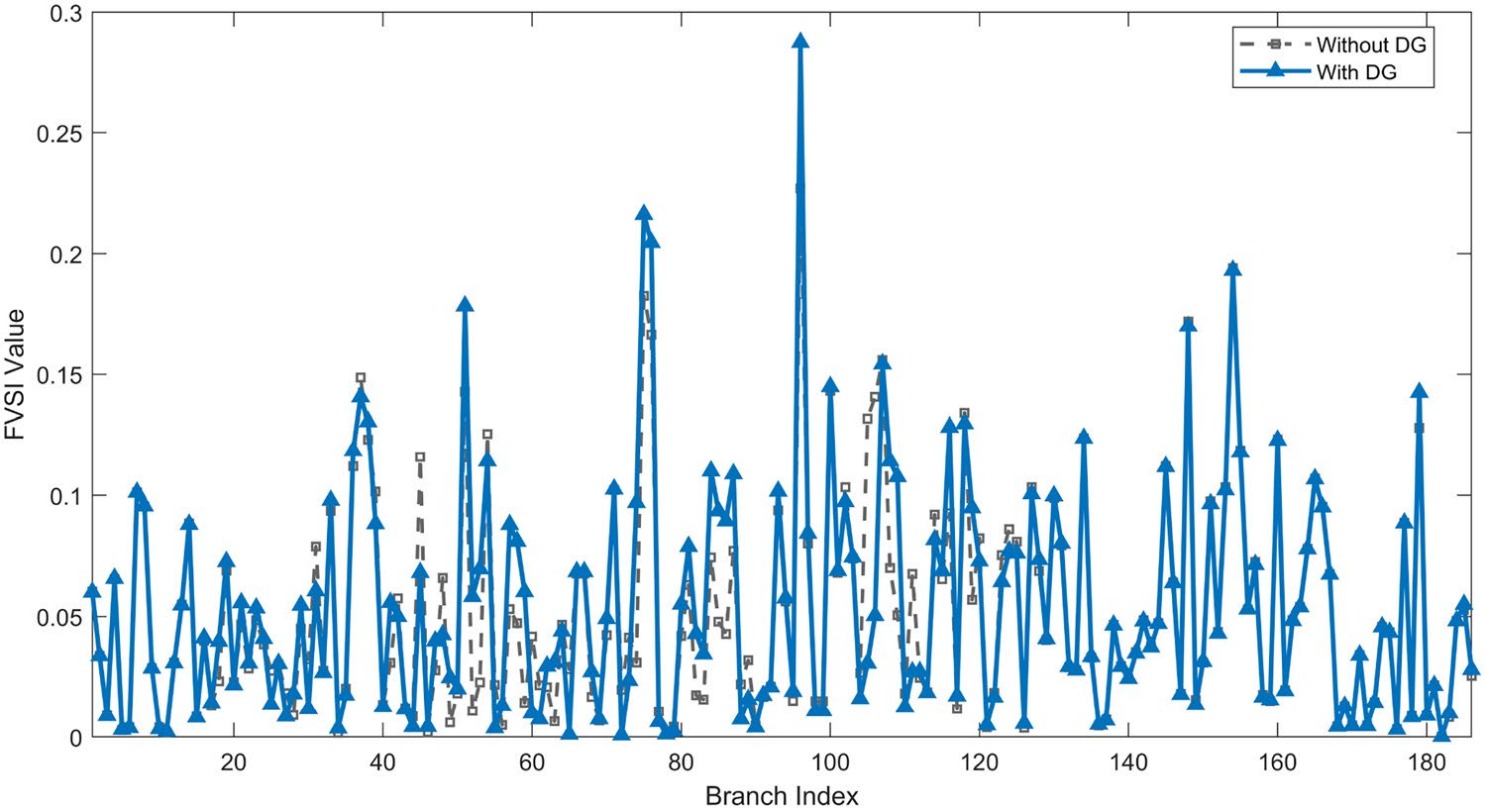
Voltage stability index comparison under normal load condition.

Under normal loading conditions, the system was analyzed to be studied in different aspects with the implementation of the three selected PVDGs:

- As shown in Fig. 23, the voltage profile of the base case was mostly stable, with a minimum voltage of 0.9430 p.u slightly below limits. By implementing the PVDG units, this minimum voltage was maintained as it is, but the overall profile became flatter indicating that the PVDGs are working well.
- The voltage lift can be quantified as we see in Fig. 24 (Voltage Lift). This graph illustrates the voltage increase at each bus using this equation $VL=V_{With DG} -V_{Without DG}$. The graph shows a maximum voltage lift of 0.047 p.u. at Bus 53. This local positive adjustment states that the reactive power support (at 0.95 PF) efficiently avoided voltage drops in this bus without violating the upper limit (1.05 p.u.)
- The VDI can show us how does the system voltage profile actually got improved. The VDI is reduced from 0.0866 in the base case to 0.0797 with the implementation of PVDG units as shown is Fig. 25, presenting a reduction in voltage deviation across all the system bus network. The Fast Voltage Stability Index (FVSI) analysis in Fig. 26 states that the system operates well within stability margins, having the value of the Max FVSI at approximately 0.28, confirming secure and safe operation under normal demand.

#### Scenario 2: light load voltage analysis

The light load condition (50% loading) is an important test for over voltage violations, and we have to make sure not to cross these limitations

- The voltage profile is robust, with the minimum voltage maintained steady at 0.9430 p.u. but making the graph flatter and more safe and secure in many buses
- Figure‘28 shows a maximum voltage increase of 0.045 p.u. at Bus 53. This incredible boost was done without violating any limits or boundaries and also ensuring system safety, showing the algorithm’s ability to balance loss reduction with voltage limitations during off-peak periods.
- The system presents high network power quality, with the VDI (Fig. 29) reduced from 0.0736 to 0.0703. The FVSI profile in Fig. 30 validates that the lines are far from instability, validating that the algorithm’s selection of the three PVDGs are holding the network system safe and secure within boundaries.

**Fig. 27:**
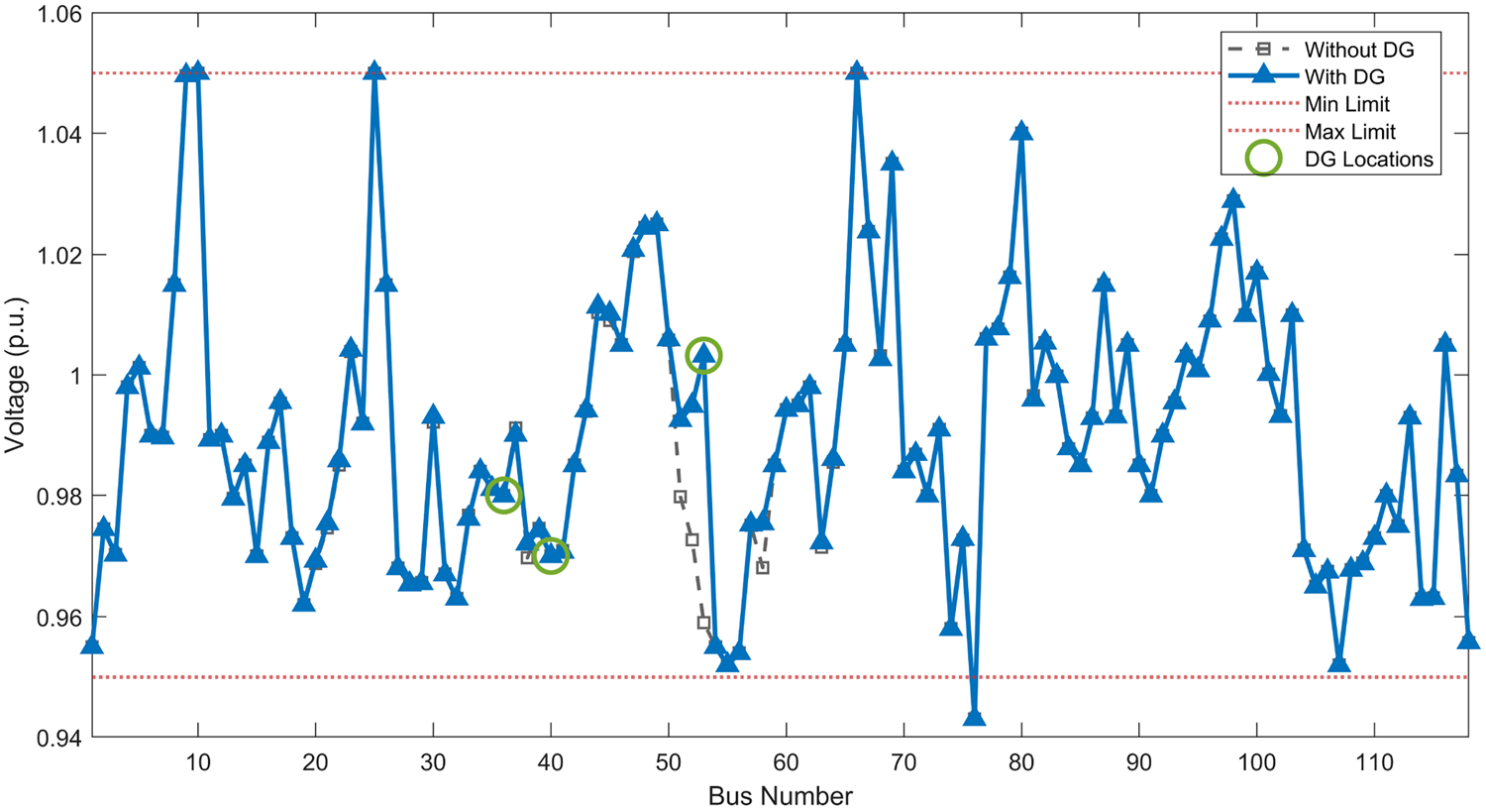
Voltage profile comparison under light load condition.

**Fig. 28:**
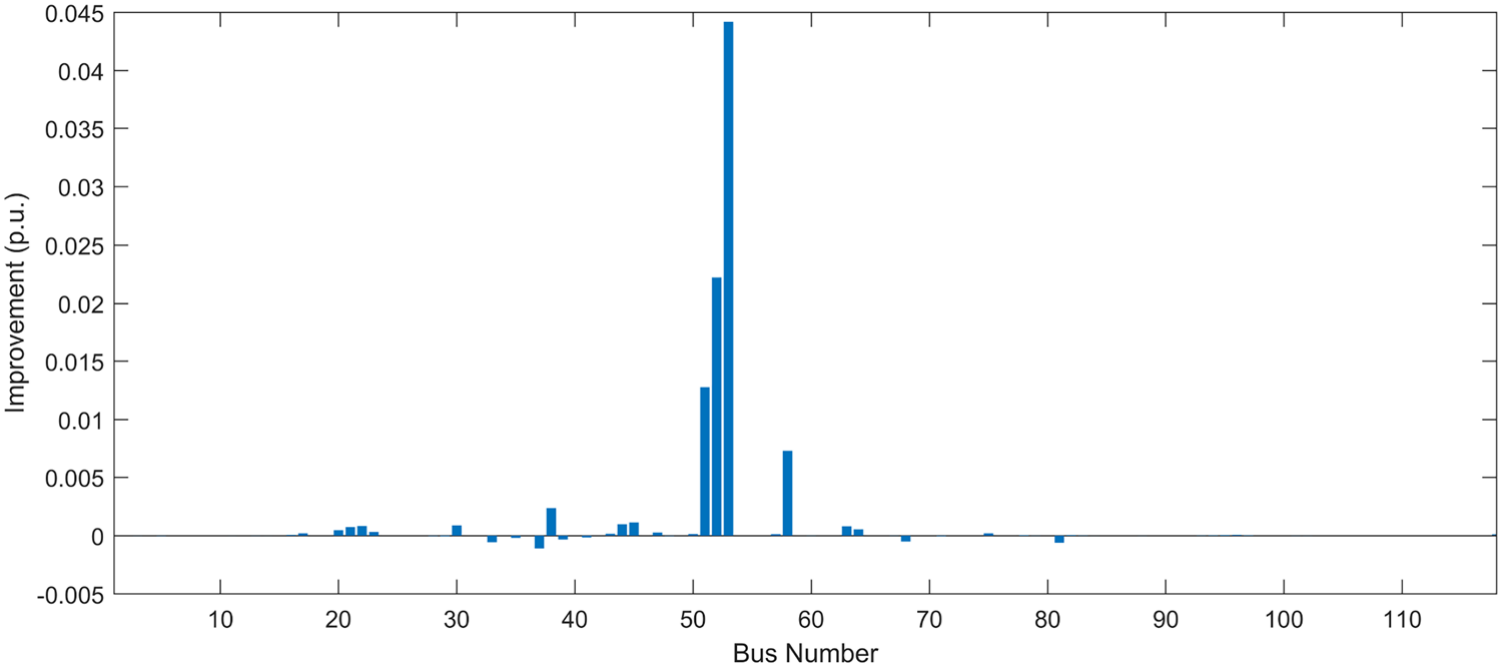
Voltage lift under light load condition.

**Fig. 29:**
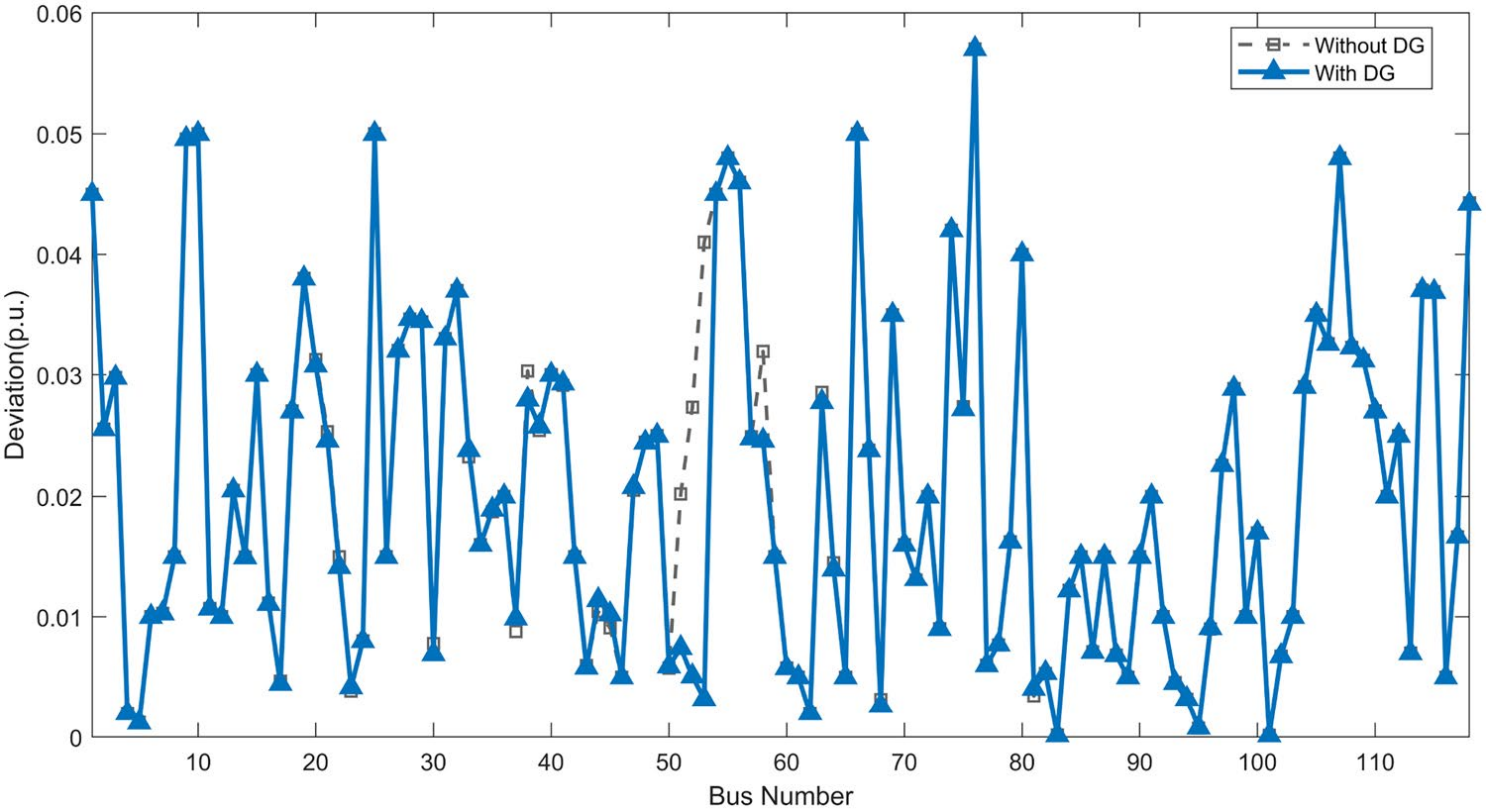
Voltage deviation index comparison under light load condition.

**Fig. 30:**
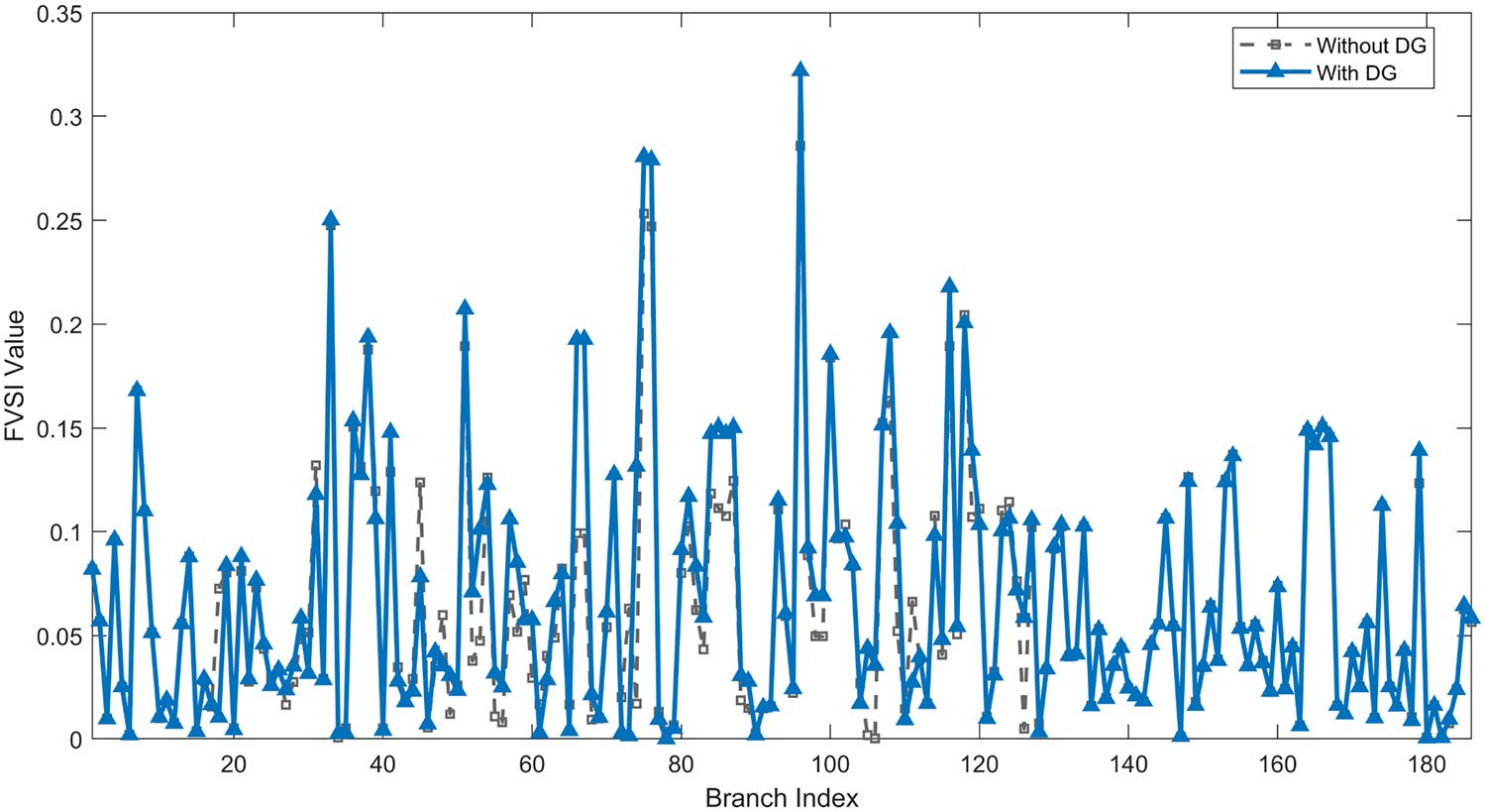
Voltage stability index comparison under light load condition.

#### Scenario 3: heavy load voltage analysis

The heavy load scenario (130% loading) put the IEEE 118-bus system in a high stress mode, really putting the system to the test to see if the PVDGs will help the system gain its stability or not.

**Fig. 31:**
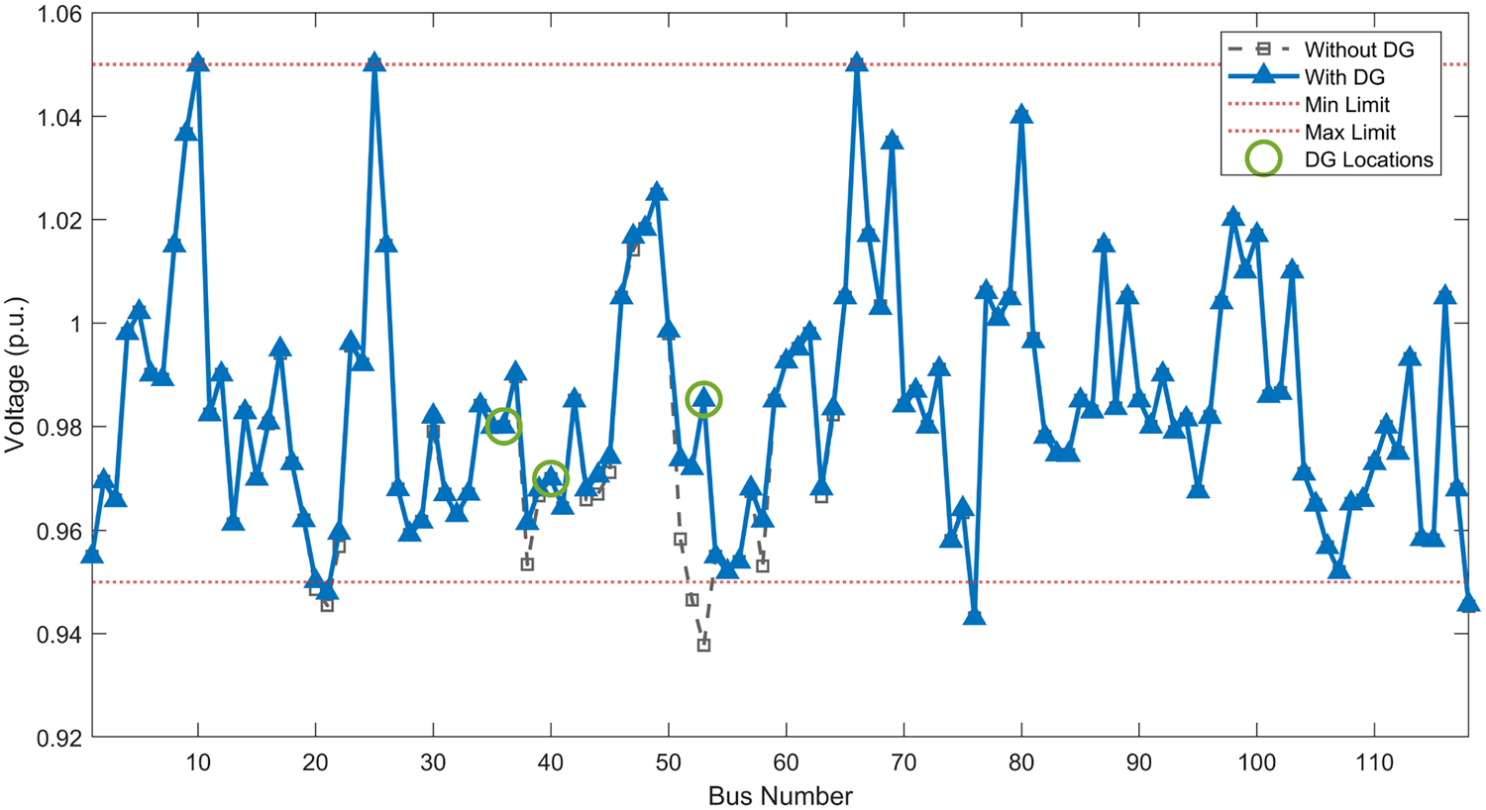
Voltage profile comparison under heavy load condition.

**Fig. 32:**
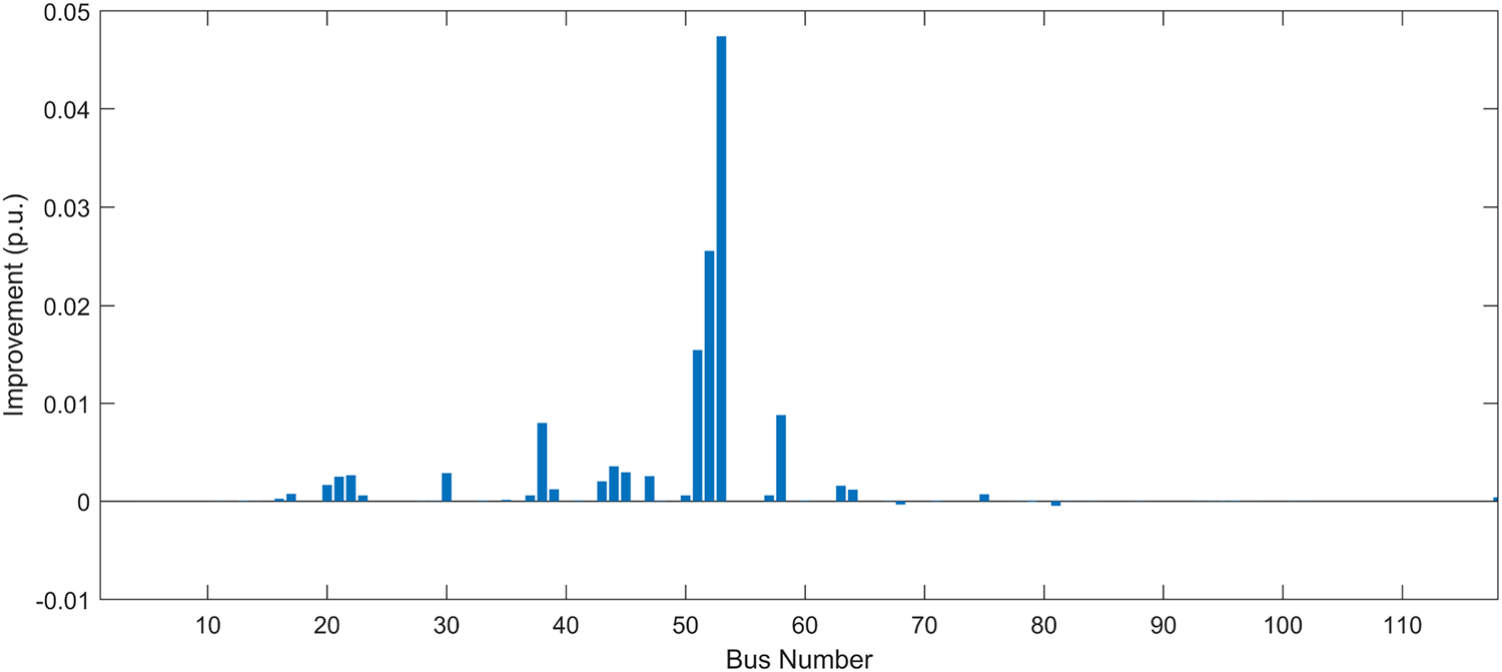
Voltage lift under heavy load condition.

**Fig. 33:**
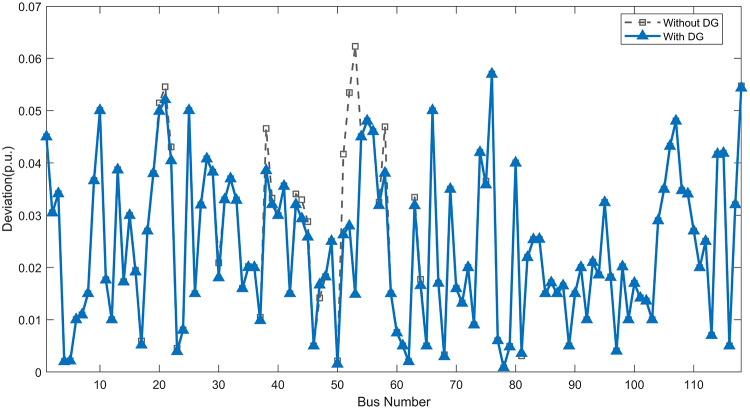
Voltage deviation index comparison under heavy load condition.

**Fig. 34:**
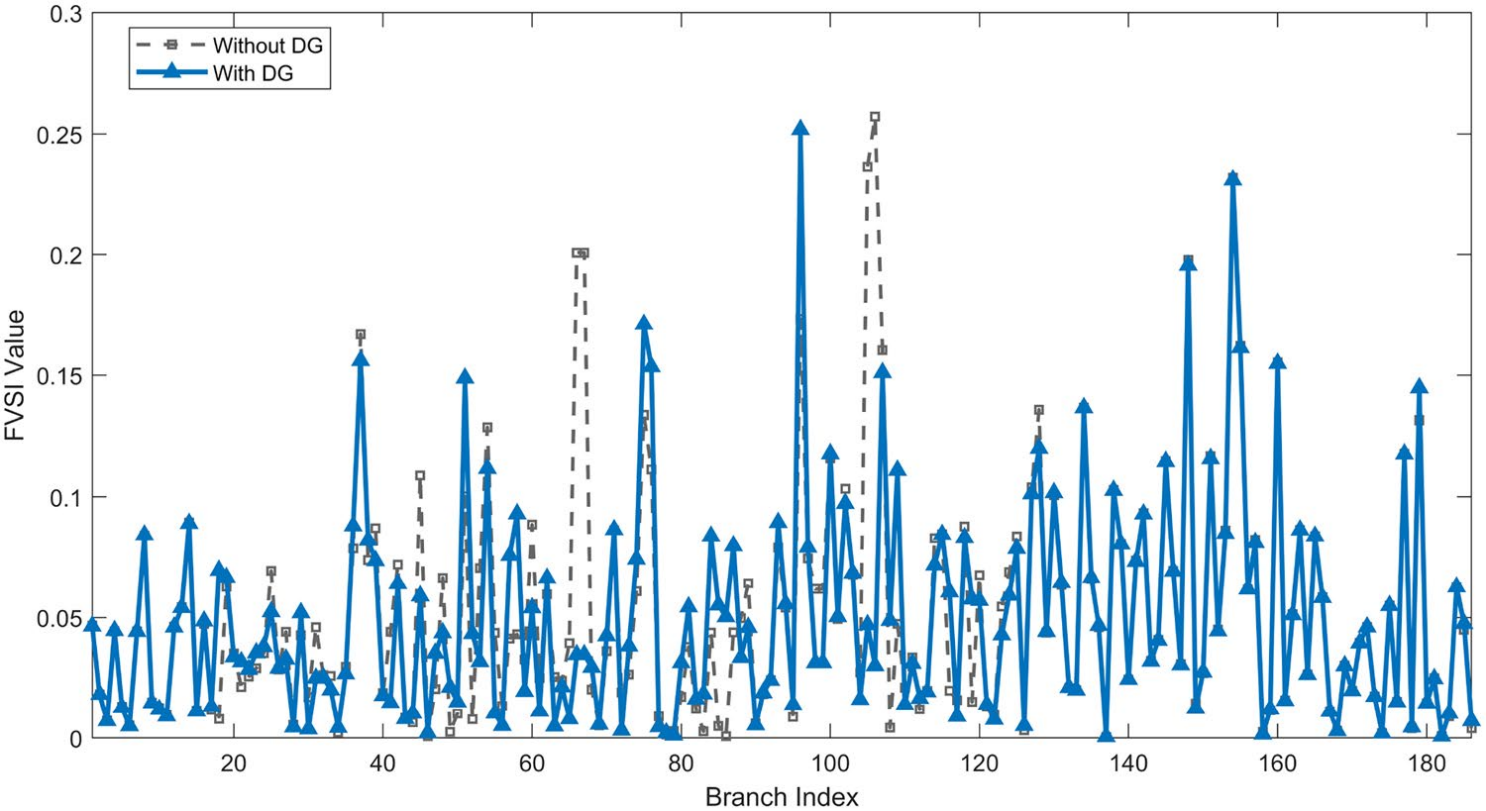
Voltage stability index comparison under heavy load condition.

- In the base case, the system faced voltage sags, with the lowest voltage at 0.9377 p.u. By implementing the selected PVDGs, this floor voltage was increased to 0.9430 p.u. Figure 31 shows us this compensation, showing how the power injection helped the system get itself to safer more secure operation condition.
- The Voltage Lift representation in Fig. 32 shows the important contribution of the PVDGs. In this scenario specifically, the voltage lift actually helped the system recover from a big voltage dive effectively acting as a counter-measure to the voltage sag associated with peak reactive power demand.
- The voltage stability improvement shown in Fig. 34 is quantified and presented by the FVSI. The maximum FVSI value in the network was reduced from 0.2572 in the base case to 0.2519 with the presence of the PVDG units. In addition, the average FVSI value across the system decreased from 0.0522 to 0.0500. The reduction in VDI shown in Fig. 33 confirms an improved flatter voltage profile.

**Fig. 35:**
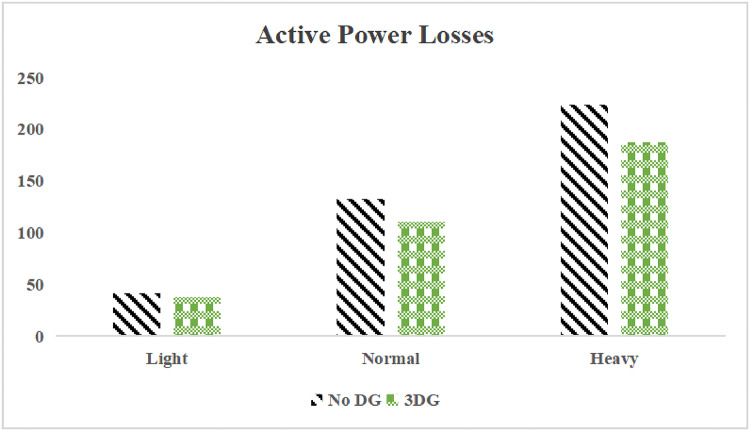
Total active power losses comparison under all loading conditions.

#### Active and reactive power losses analysis

The smart implementation of the three PVDGs across this complex system resulted in noticed improvements in the transmission network’s efficiency. This part shows the reduction in active and also reactive power losses, starting with a comparison between base case and the use of our PVDGs, and then a thorough representation of power losses comparison across each branch.

**Fig. 36:**
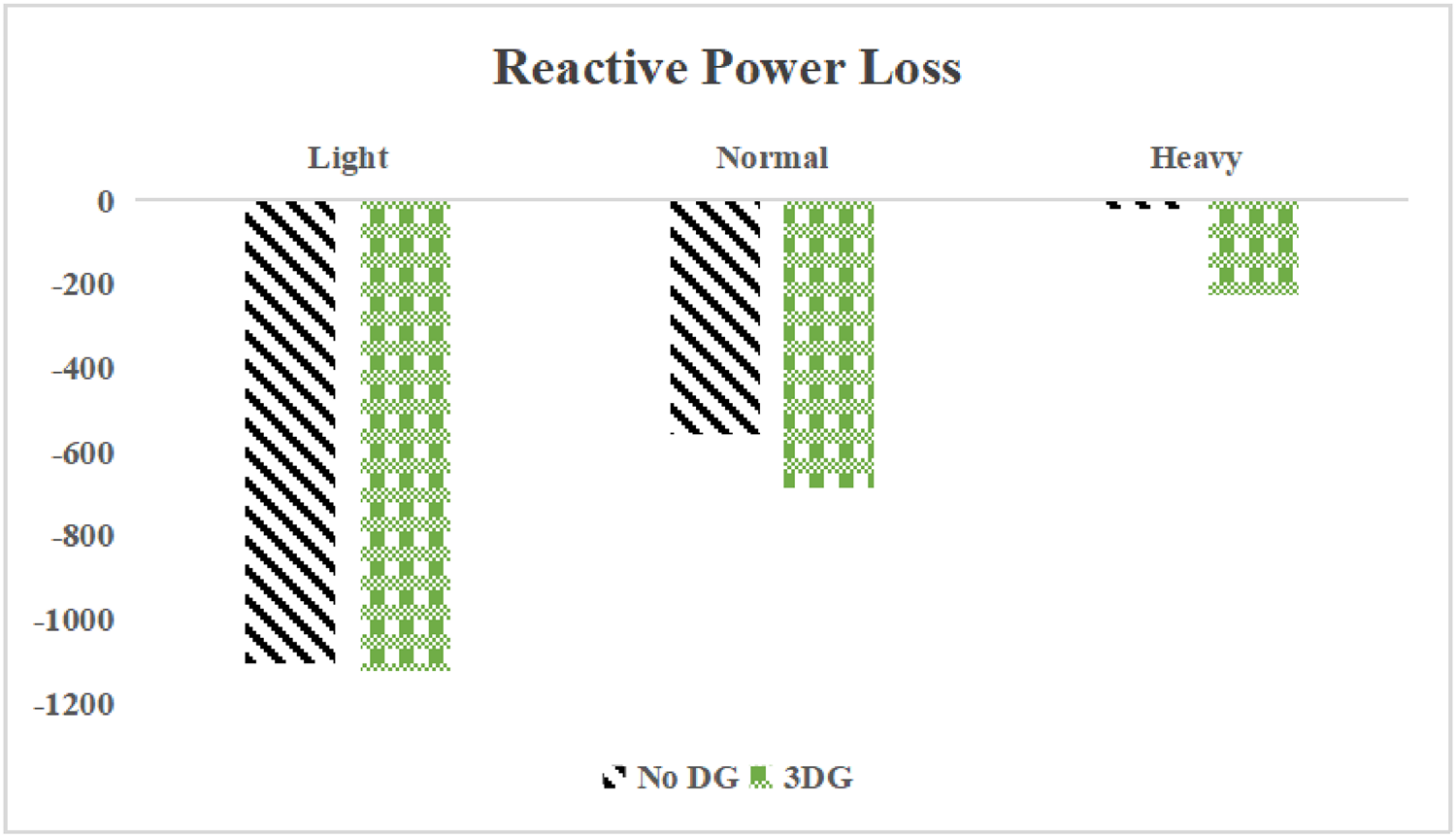
Total reactive power losses comparison under all loading conditions.

**Fig. 37:**
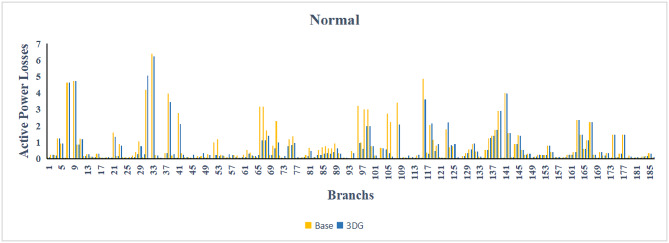
Active power losses across all branches in normal load condition.

**Fig. 38:**
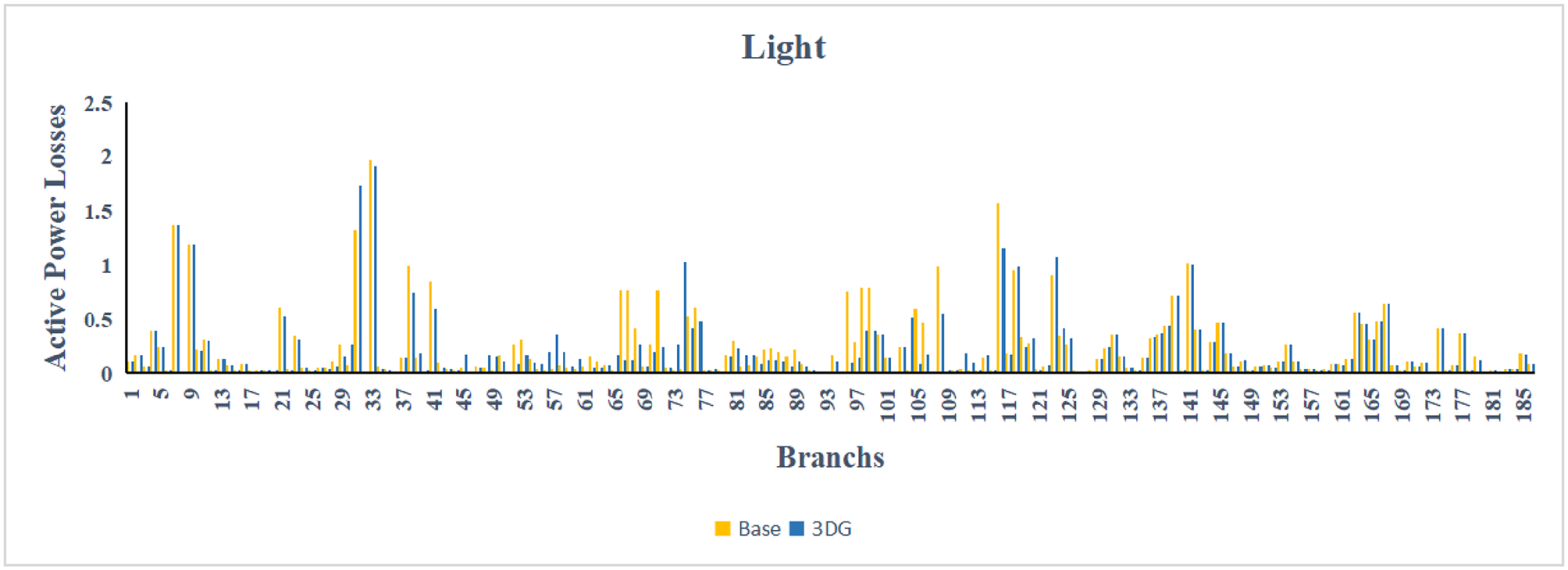
Active power losses across all branches in light load condition.

**Fig. 39:**
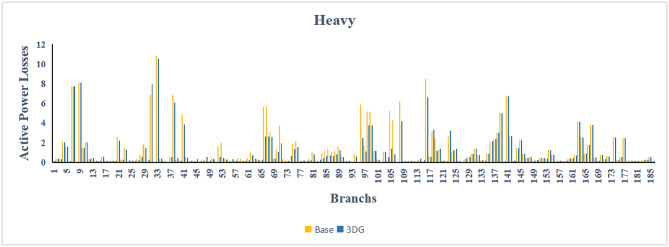
Active power losses across all branches in heavy load condition.

- Total Active and Reactive Power Loss Reduction Fig. 35, 36 provide a full perspective of the total power losses across all three loading scenarios
  - Normal Load: The total active power losses were reduced from 132.86 MW to 110.30 MW, representing a 16.98% reduction and save 22.56 MW in active power.
  - Light Load: The system active power losses were reduced from 41.23 MW to 37.77 MW representing a 8.39% reduction and save 3.46 MW in active power.
  - Heavy Load: The most power saving can be found in heavy load scenario, where losses were reduced from 224.01 MW to 187.64 MW. This 16.24% reduction save over 36.37 MW
  The implementation of the decided three PVDGs reduced stress on the system and reduced the reactive power losses across the system as shown in Fig. 36.
  - Normal Load: Total reactive losses were reduced from −557.95 MVar to −684.84 MVar.
  - Light Load: Total reactive power losses were reduced from −1104.09 MVar to −1124.15 MVar.
  - Heavy Load: A massive reduction was seen, from −21.48 MVar to −226.91 MVar. This massive decrease proving that the PVDG units successfully compensated for the $I^2*X$ drops in the network, leaving space for active power transfer.
- B.Branch-wise Loss Minimization To understand the specific impact on network congestion, the losses were analyzed on a branch-by-branch basis. Figures 37, 38, 39 visualize the active power losses for the network branches. In the uncompensated base case, critical transmission corridors connecting major generation hubs to load centers exhibited high resistive losses due to heavy power transfers. Unlike the radial distribution system, the losses here are distributed across loops. Following the optimal allocation of the PVDG units at Buses 36, 40, and 53, the power flow was redistributed locally. This injection reduced the current magnitude flowing through the long-distance transmission lines, effectively unloading the congested branches. Consequently, the active power losses in these high-traffic branches dropped noticeably, as evidenced by the ”With DG” bars.

**Fig. 40:**
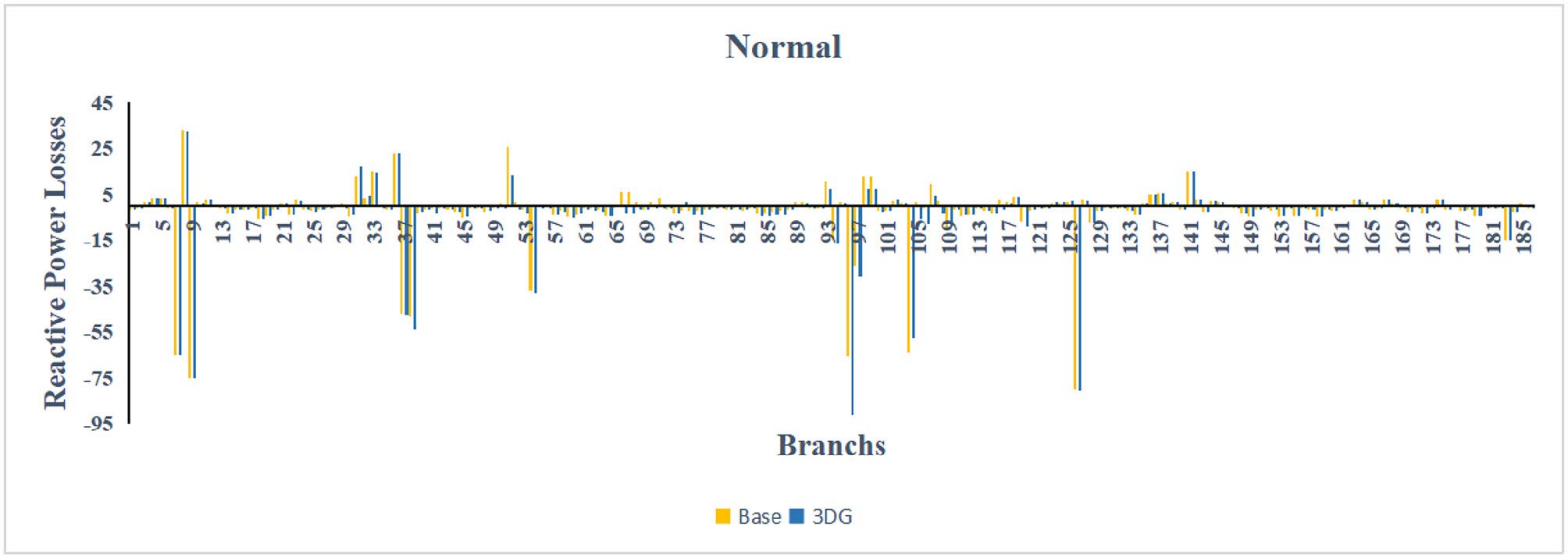
Reactive power losses across all branches in normal load condition.

**Fig. 41:**
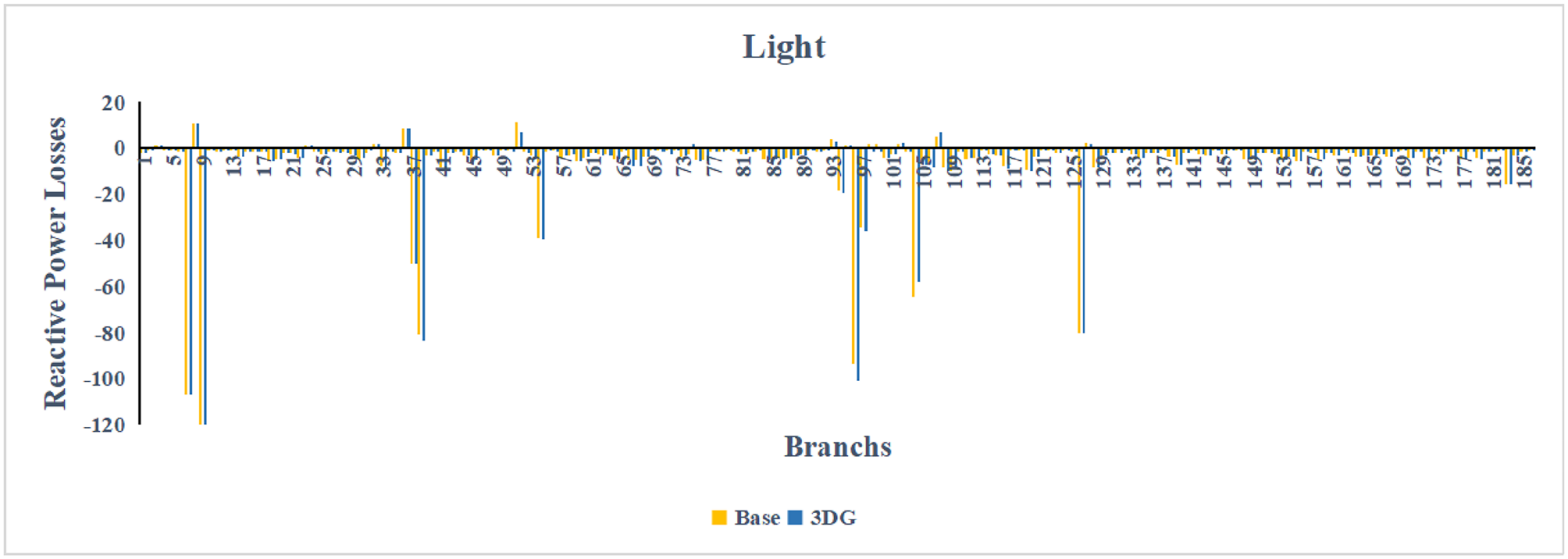
Reactive power losses across all branches in light load condition.

**Fig. 42:**
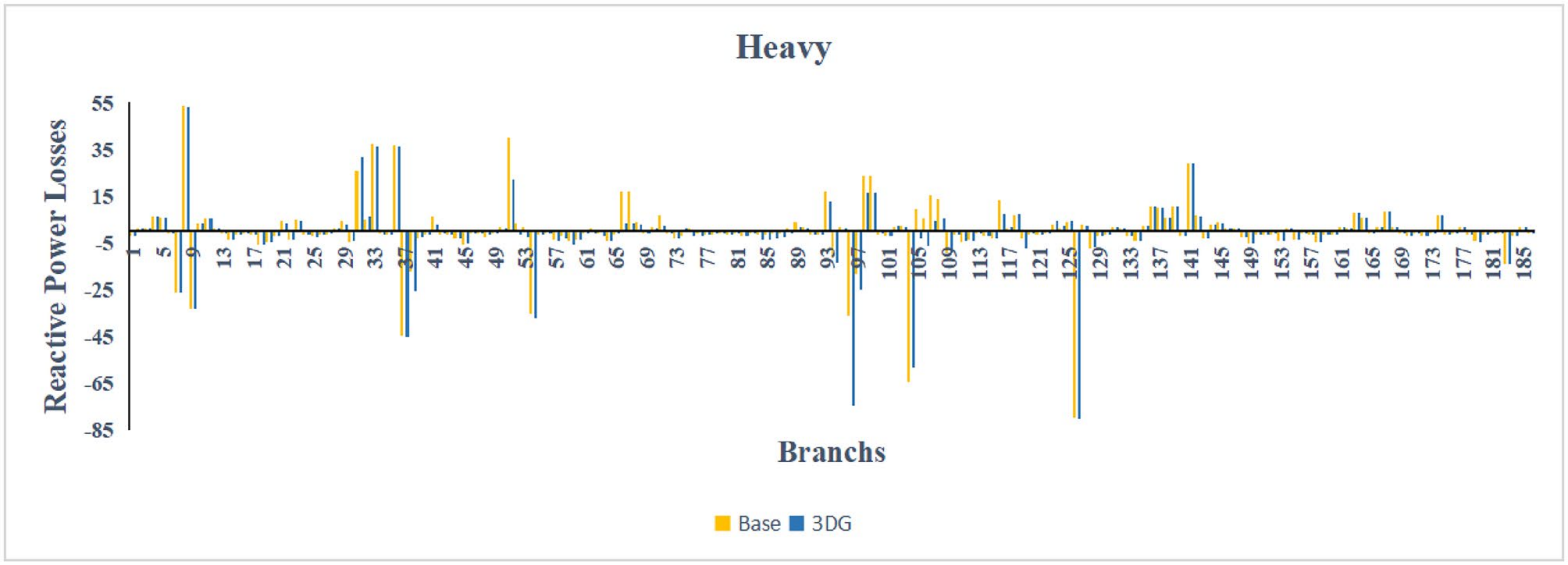
Reactive power losses across all branches in heavy load condition.

A similar trend is observed for reactive power losses in Figures 40, 41 and 42. The distributed generation effectively supplied a portion of the local reactive demand (operating at 0.95 PF), reducing the reactive current component flowing through the transmission lines. This drastically minimized the reactive voltage drops across the network and alleviated the burden on the central synchronous generators. This local relief mechanism is a primary driver behind the global performance enhancement of the IEEE 118-bus transmission system.

### IEEE 118-bus transmission system results (Scenario B: Hybrid)

This section presents the simulation results for the complex IEEE 118-bus transmission system. The (DOA) was employed to find the optimal placement of hybrid renewable energy sources.

#### Optimal sizing and placement

The DOA algorithm selected six buses for the implementation of the hybrid renewable energy system. The algorithm uses a simultaneous 12 variable optimization approach. This allowed for the parallel determination of both the size and location for all six DG units (3 PV and 3 Wind) within a single solution vector. The objective function has really strict constraints to ensure technical practicality, including a smart location check to prevent double placement on the same bus and exclude the slack bus number 69 from the options. The optimal formation was obtained by minimizing the cumulative weighted active power losses across Light, Normal, and Heavy loading scenarios ($W_{Light} = 0.2, W_{Normal}=0.6, W_{Heavy}=0.2$), while avoiding any voltage violations outside the 0.95–1.05 p.u. permissible range. Also, we set an optimal power factor of 0.95 to all DGs as a real life representation of needed reactive power for inverters and to support the local voltage profile. The location and sizes of all DGs implemented in the system are as represented in Table 6.

**Table 6:** Optimal locations and corresponding capacities determined by the DOA.

Bus	Power (MW)
35	154.63
40	97.24
45	80.36
53	165.93
105	165.85
118	66.66
**Total Power**	**729.67 MW**

#### Scenario 1: normal load voltage analysis

**Fig. 43:**
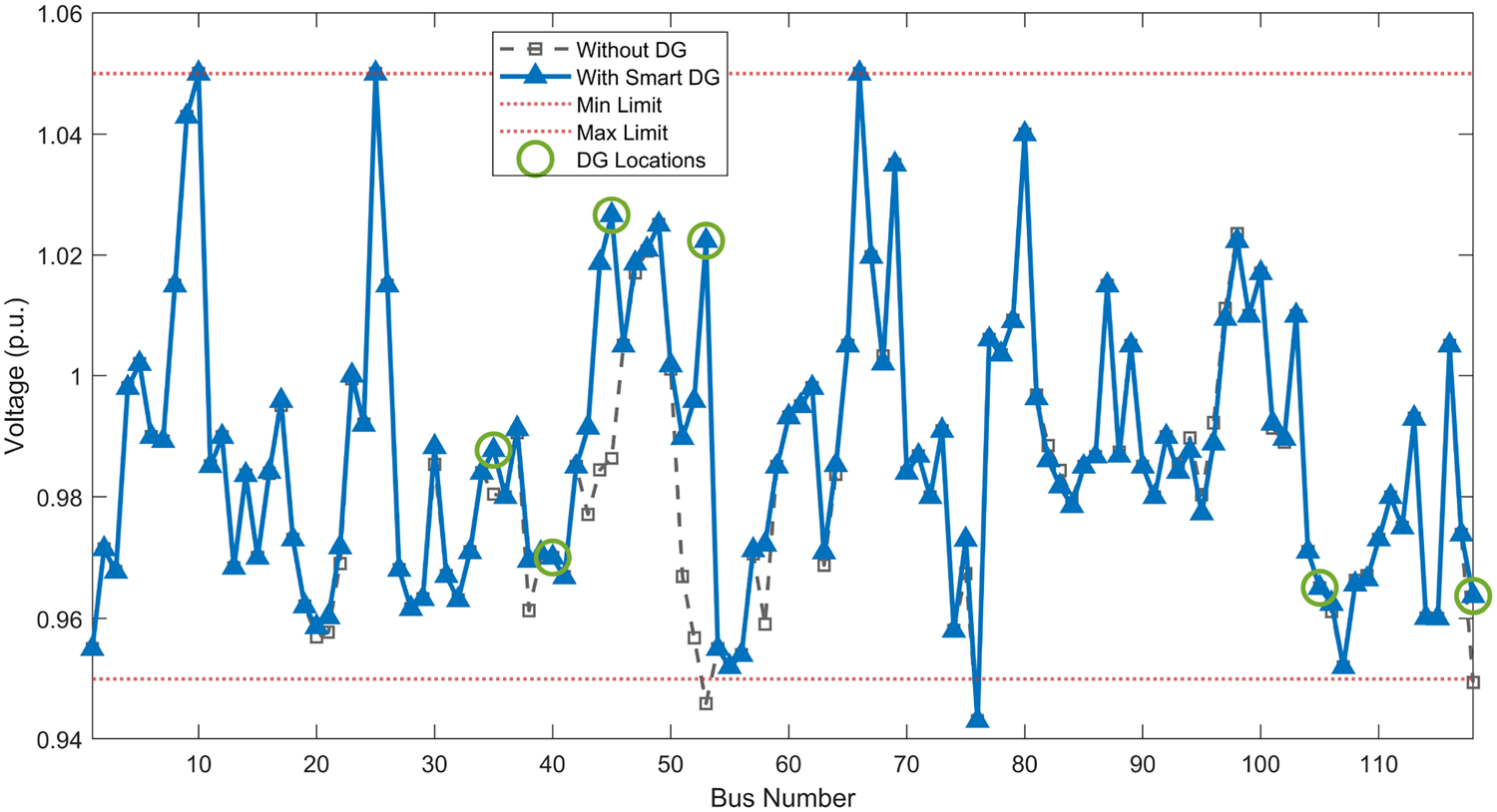
Voltage profile comparison under normal load condition.

**Fig. 44:**
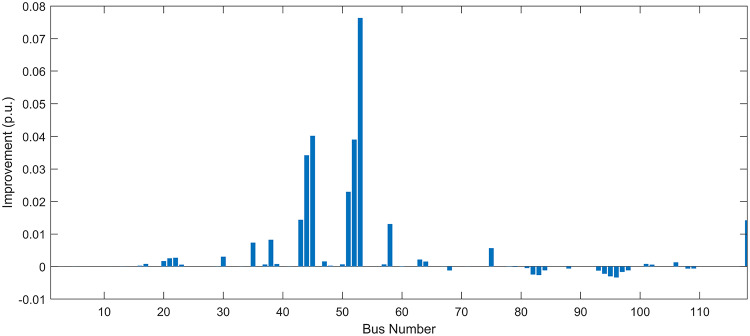
Voltage lift under normal load.

**Fig. 45:**
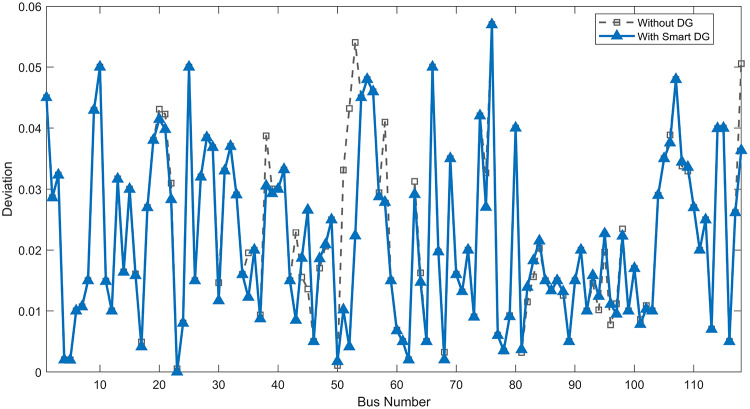
Voltage deviation index comparison under normal load condition.

**Fig. 46:**
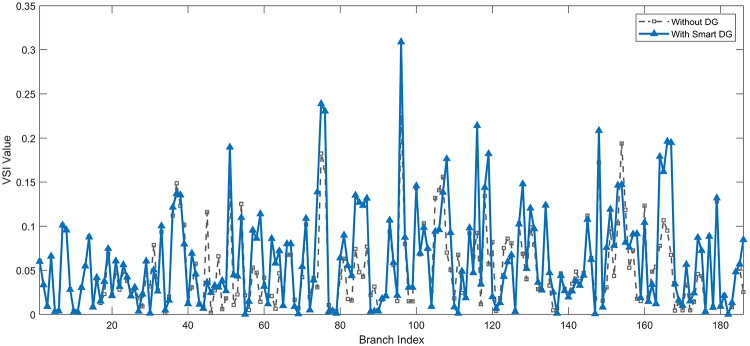
Voltage stability index comparison under normal load condition.

Under normal loading conditions, the system was analyzed to be studied in different aspects with the implementation of all assigned DGs:

- Voltage Profile: the base case voltage profile as shown is Fig. 43 have its deepest dive in voltage value of 0.943 p.u. Even after introducing the hybrid system, the lowest voltage value remain 0.943. Despite that, the overall voltage profile graph is flatter, holding the system secure and safer that the base case.
- Voltage Lift: The contribution of the DGs can be quantified in the Voltage Lift analysis (Fig. 44). This graph shows the net voltage increase at each bus ($V_{WithDG}-V_{base}$). The analysis presents a maximum Voltage Lift (VL) of 0.076 p.u. at Bus 53. This boost indicates that the hybrid system is successfully working in compensating to the voltage drops across the system while maintaining the safety of the system within the margin and voltage doesn’t exceeds 1.05 p.u.
- The improvement in the network quality is quantifiable by the VDI and the FVSI. The VDI is reduced from 0.0866 in the base case to 0.0778 with the use of DG units shown in Fig. 45, confirming a safer, more stable system. The Fast Voltage Stability Index shown in Fig. 46 confirms that the system works great within stability margins, the maximum FVSI calculated at 0.3087 below the critical limit of 1.0, ensuring secure operation under normal loading.

#### Scenario 2: light load voltage analysis

In the Light Loading scenario, a new challenge was seen where low demand caused a reverse power flow and the injection of DGs made the power losses increase. To counter this challenge, a Dispatch Factor was introduced to scale the DG output, ensuring technical applicability.

**Fig. 47:**
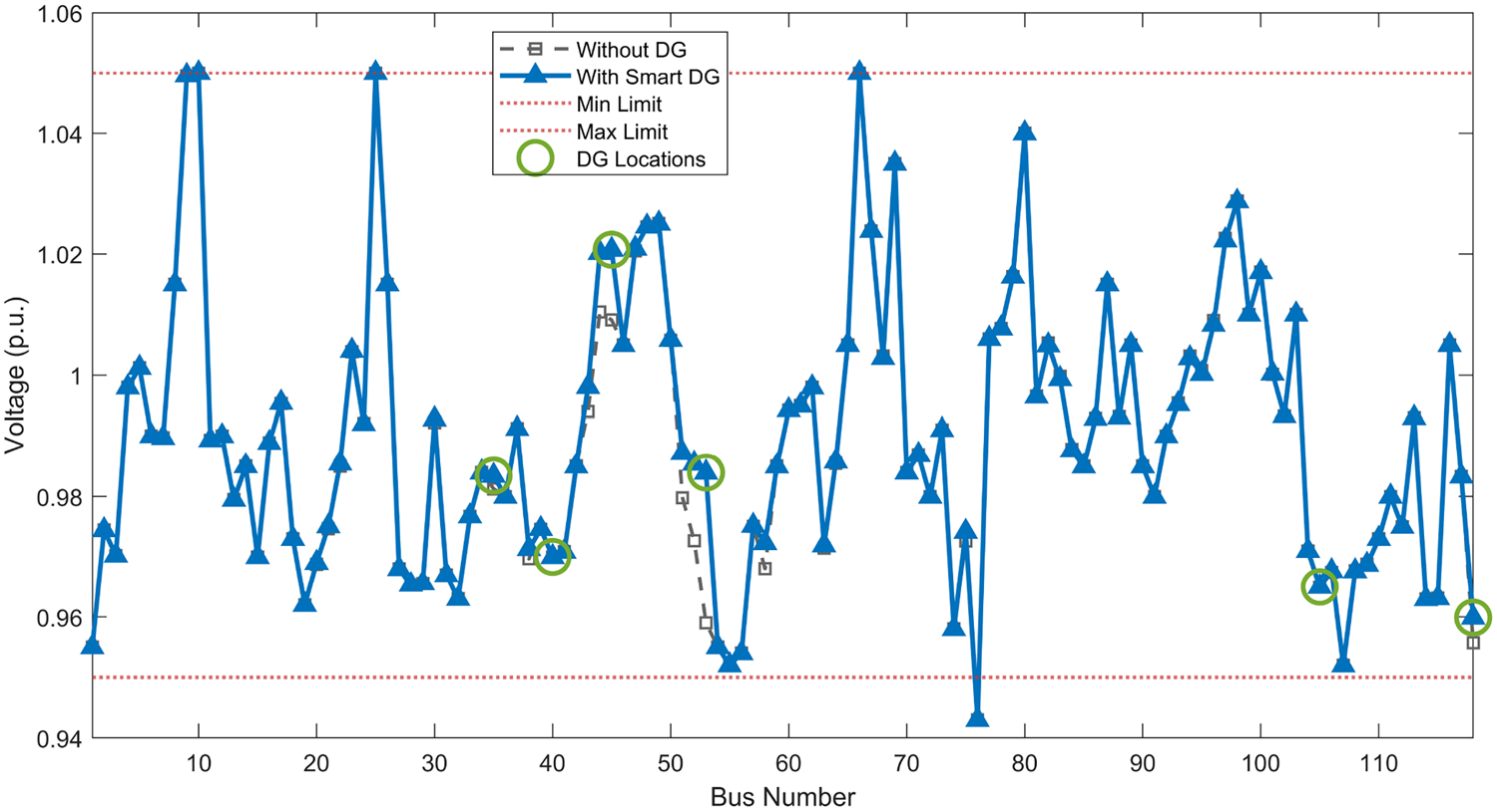
Voltage profile comparison under light load condition.

**Fig. 48:**
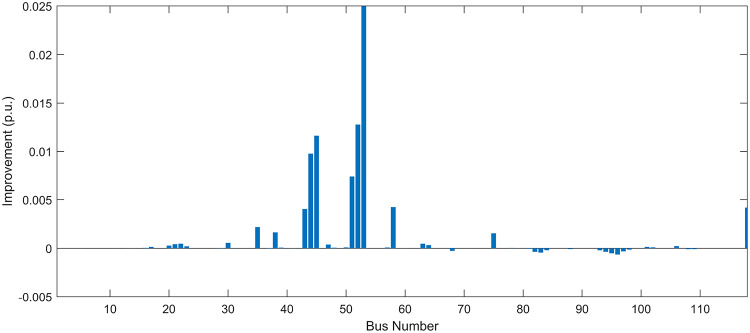
Voltage lift under light load condition.

**Fig. 49:**
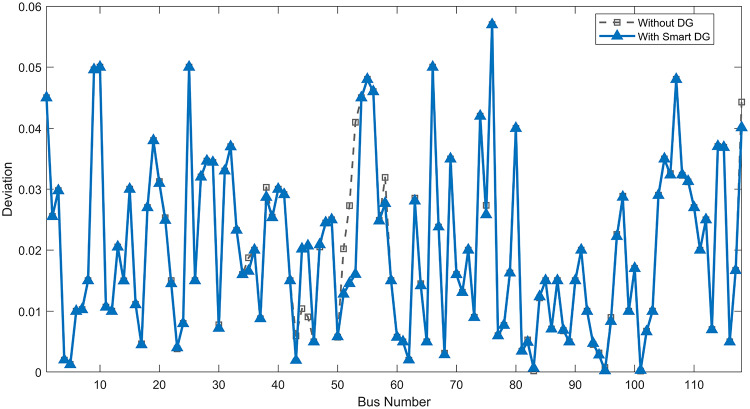
Voltage deviation index comparison under light load condition.

**Fig. 50:**
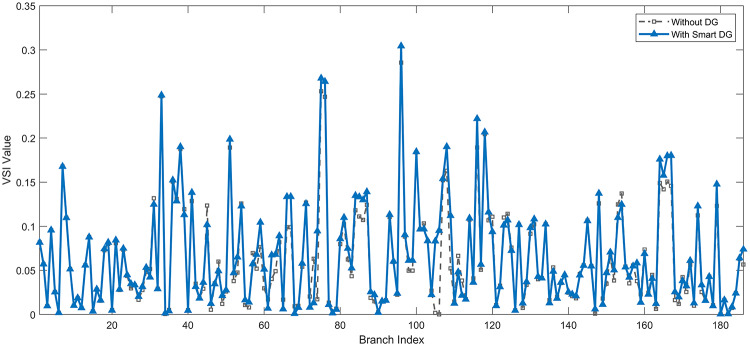
Voltage stability index comparison under light load condition.

- Voltage Profile: With the Dispatch factor in hand, as shown in Fig. 47, the lowest point in the voltage profile in the base case was 0.943 p.u. When applying the DGs the lowest point remains the same but the graph becomes flatter and more stable and have less ripples. The use of the dispatch factor avoided over voltage problems that happened from full DG injection in low demand scenarios, making sure the voltage remained within the acceptable limits.
- Voltage Lift: Even with the reduced DGs output limited by the dispatch factor, the system performs better with voltage support of the DG units. The maximum Voltage Lift seen was 0.025 p.u. at Bus 53 shown in Fig. 48. This lift proves that the DGs still provide good support to needed buses without putting the grid in over voltage issues and still respecting the system’s boundaries.
- Stability Indices: The power quality experienced significant improvement, with the VDI reduced from a value of 0.0736 to 0.0710, shown in Fig. 49. This reduction confirms that even with the dispatch factor making the DGs power output scaled to 0.3, the DGs contributed to a better, more stable voltage profile. The system stability, shown in Fig. 50, is within the secure and stable margins with a maximum FVSI of 0.3044, showing that the dispatch strategy successfully handled power loss reduction with system integrity and security.

#### Scenario 3: heavy load voltage analysis

With Heavy Loading scenario, the system is under maximum stress. The selected DGs performed significantly well, providing better system safety and getting the system to operate away of instability and stress.

**Fig. 51:**
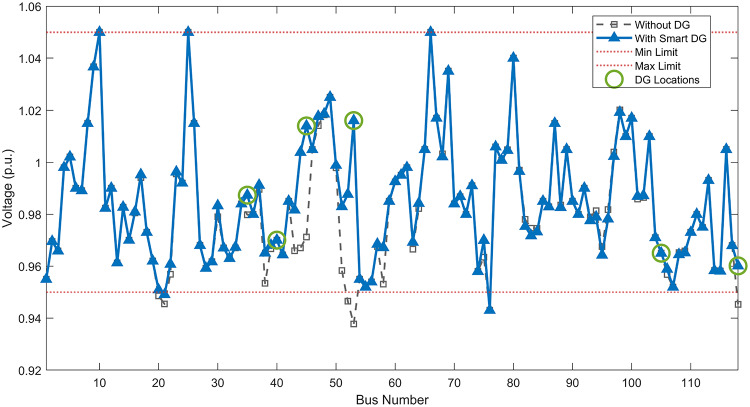
Voltage profile comparison under heavy load condition.

**Fig. 52:**
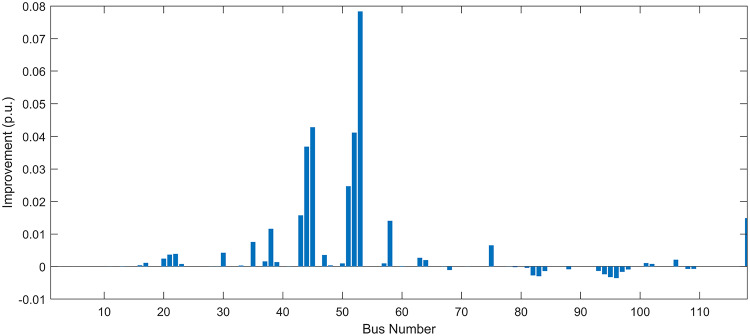
Voltage lift under heavy load condition.

**Fig. 53:**
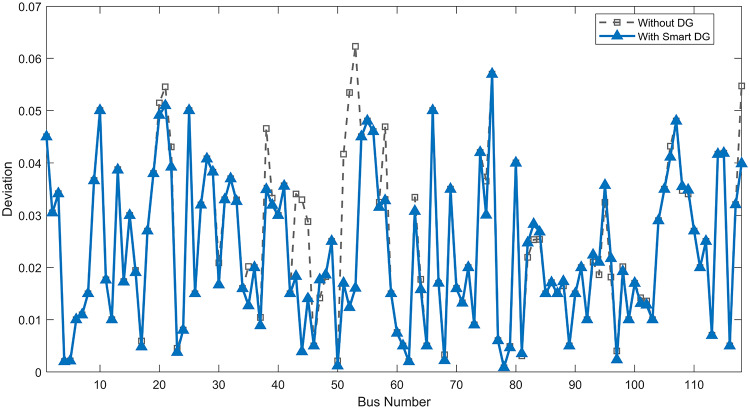
Voltage deviation index comparison under heavy load condition.

- Voltage Profile: As shown in Fig. 51, the minimum voltage is increased significantly from a dangerous value of 0.9377 p.u. in the base case to a safer 0.9430 p.u. in the presence of the DGs. This enhancement successfully dragged the system away from danger at lower voltage, avoiding violating limits during peak demand.
- Voltage Lift: The system experienced a significant voltage boost, with a maximum Voltage Lift of 0.078 p.u. seen at Bus 53 (Fig. 52). This big lift value shows that the DGs played an important role in relieving the stress on the transmission lines, injecting power where the system needs it to ensure the quality and the safety of the system within its permissible range.
- Stability Indices: The Voltage Deviation Index shows a significant improvement as shown in Fig. 53, reduced from value of 0.1015 to 0.0863. This massive reduction can be shown in a flatter, much more uniform voltage profile across the complex system. Also, the maximum FVSI shown in Fig. 55 was calculated at the value of 0.2817, confirming that the hybrid DGs implementation in the system successfully improve the voltage stability even when it’s under a massive stress in heavy load scenario.

**Fig. 54:**
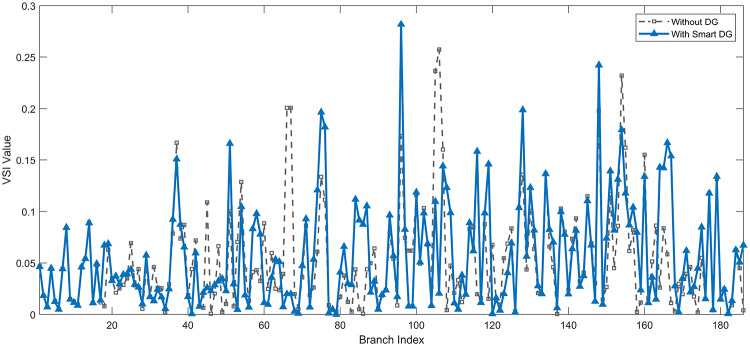
Voltage stability index comparison under heavy load condition.

#### Active and reactive power loss analysis

The strategic usage of the six DOA optimized hybrid units resulted in remarkable enhancement in our transmission network’s efficiency and safety. This section visualize the power reduction in both active and reactive power losses, starting with a comparison between base case and the use of our DGs, and then a thorough representation of power losses comparison across each branch.

**Fig. 55:**
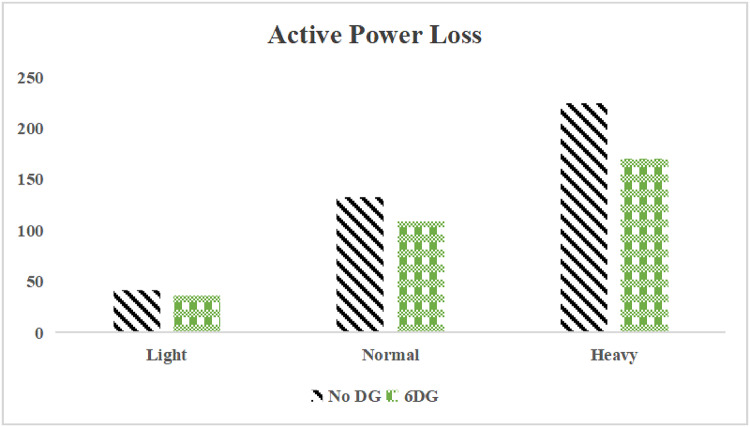
Total active power losses comparison under all loading conditions.

**Fig. 56:**
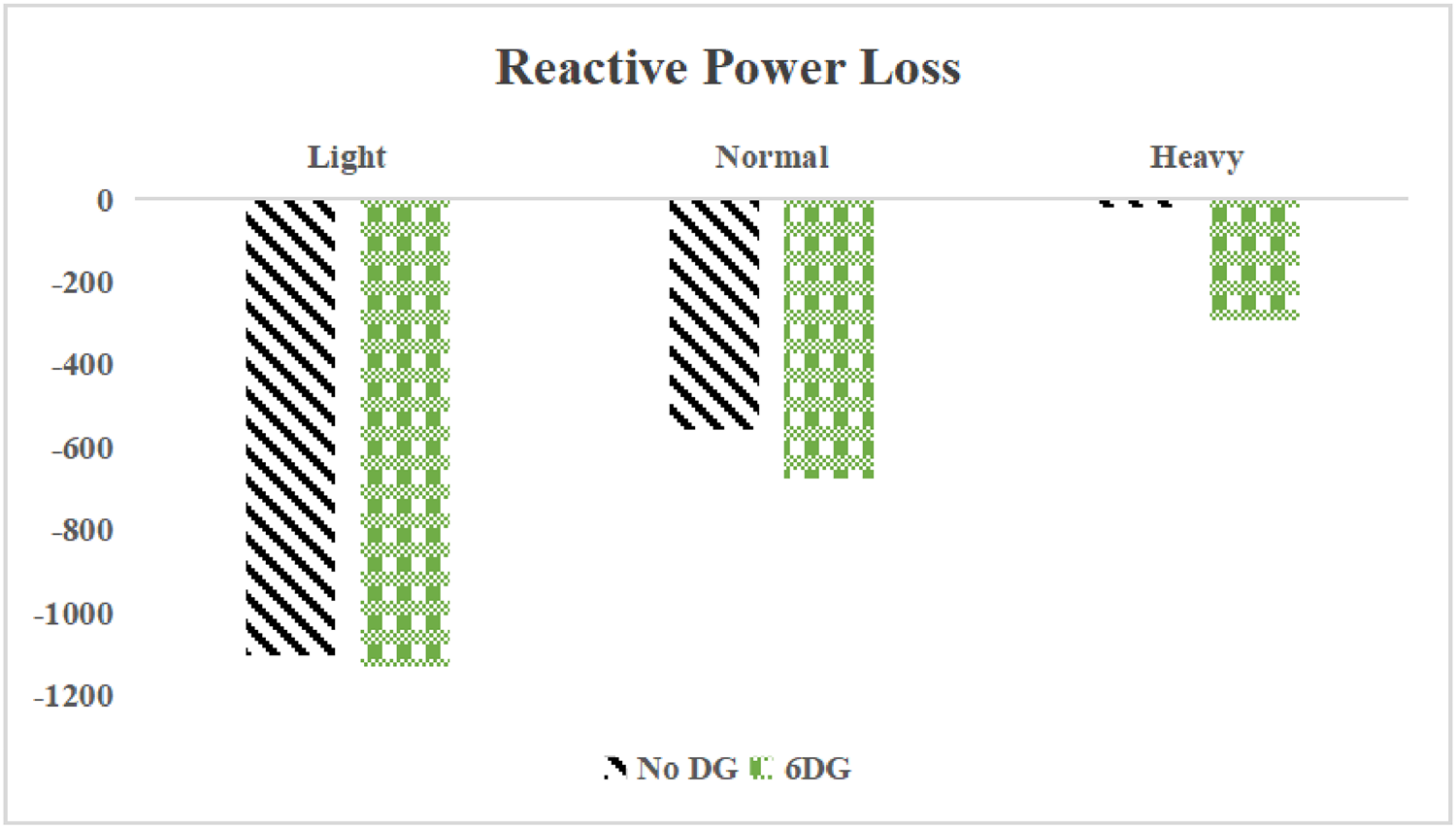
Total reactive power losses comparison under all loading conditions.

**Fig. 57:**
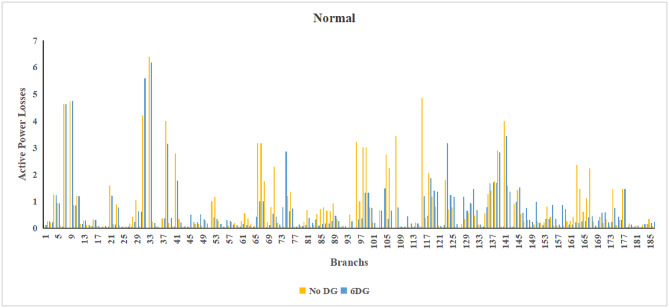
Active power losses across all branches in normal load condition.

**Fig. 58:**
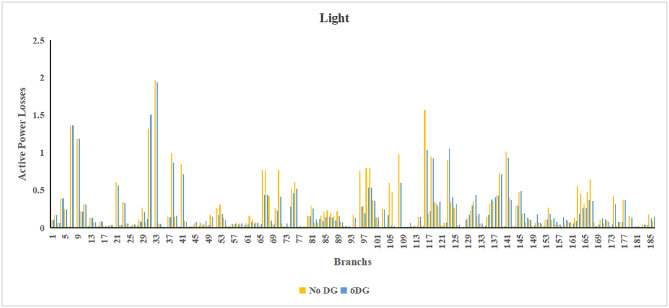
Active power losses across all branches in light load condition.

**Fig. 59:**
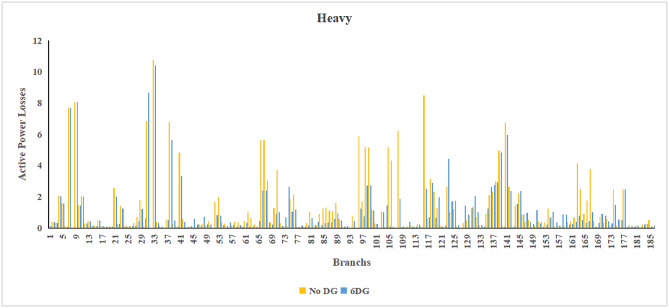
Active power losses across all branches in heavy load condition.

- Total Active and Reactive Power Loss Reduction Fig. 55, 56 provide a complete clear perspective of the total power losses across all three loading scenarios.
  - Normal Load: The total active power loss was reduced from 132.86 MW to 108.85 MW, representing an 18.07% reduction.
  - Light Load: Initially, we simulated the power losses without the Dispatch factor and we got a results of negative percentage (−42.07%) of power loss reduction. That means that we increased system losses due to reverse flow. However, after implementing the smart idea of the Dispatch Factor (0.3) to scale the DG output, the system performance was changed to a positive percentage as we expect. The final result achieved a reduction from 41.23 MW to 36.03 MW, securing a 12.61% improvement and save 5.2 MW in power losses.
  - Heavy Load: Having the most power demand means we can get the most power losses reduction using the DGs. Total active power losses is reduced from a value of 224.01 MW to 170.28 MW. This 23.98% reduction saving over 53 MW in active power removing stress from major transmission lines when the grid is most vulnerable to failure.
  Reactive Power: The use of the DGs proved significant reaction power relief across the system. As shown in Fig. 56, the total reactive losses calculated by this equation went deeper into the negative range, proving reduced series reactive consumption across the lines. Fig. 56.
  - Normal Load: Total reactive power losses decreased from −557.95 MVar to −676.73 MVar.
  - Light Load: The trend continued under light loading, with losses changing from −1104.09 MVar to −1131.16 MVar
  - Heavy Load: A massive change was observed, getting from value of −21.48 MVar to −292.84 MVar. This massive change indicates that the hybrid DG units implementation successfully lowered the current flow, hence significantly reducing the $I^2*X$ losses in the transmission lines and freeing up capacity for active power transfer.
- B.Branch-wise Loss Minimization To better see and visualize the reduction in power losses, we should see the details more thorough representation of power losses across each branch. Figures 57, 58 and 59 visualize the active power losses for the network branches. In the uncompensated base case, critical transmission corridors connecting major generation hubs to load centers exhibited high resistive losses due to heavy power transfers. Following the optimal allocation of the hybrid units at Buses 105, 40, 35, 45, 53, and 118, the power flow was redistributed locally. This dispersed injection reduced the current magnitude flowing through the long-distance transmission lines, effectively unloading the congested branches. Consequently, the active power losses in these high-traffic branches dropped noticeably, as evidenced by the ”With DG” bars.

**Fig. 60:**
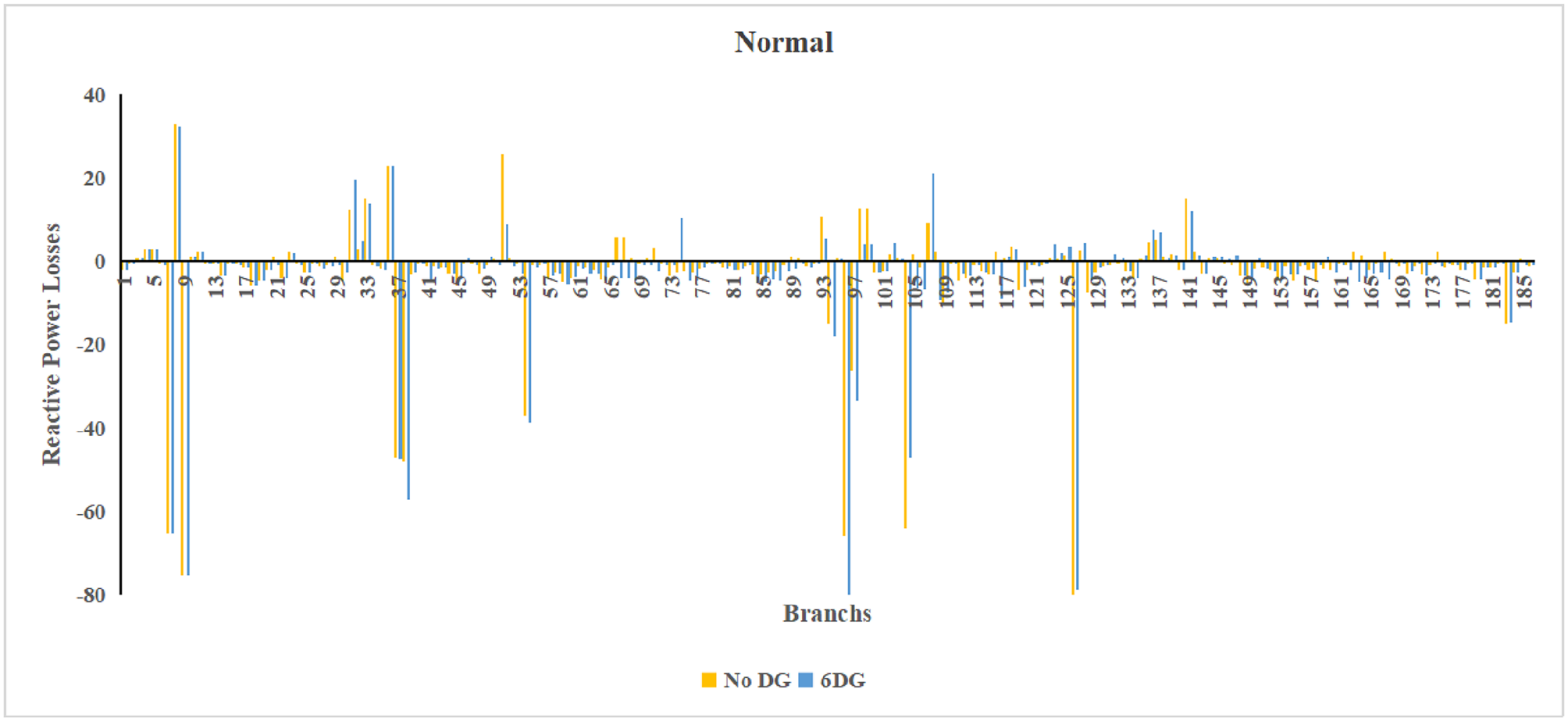
Reactive power losses across all branches in normal load condition.

**Fig. 61:**
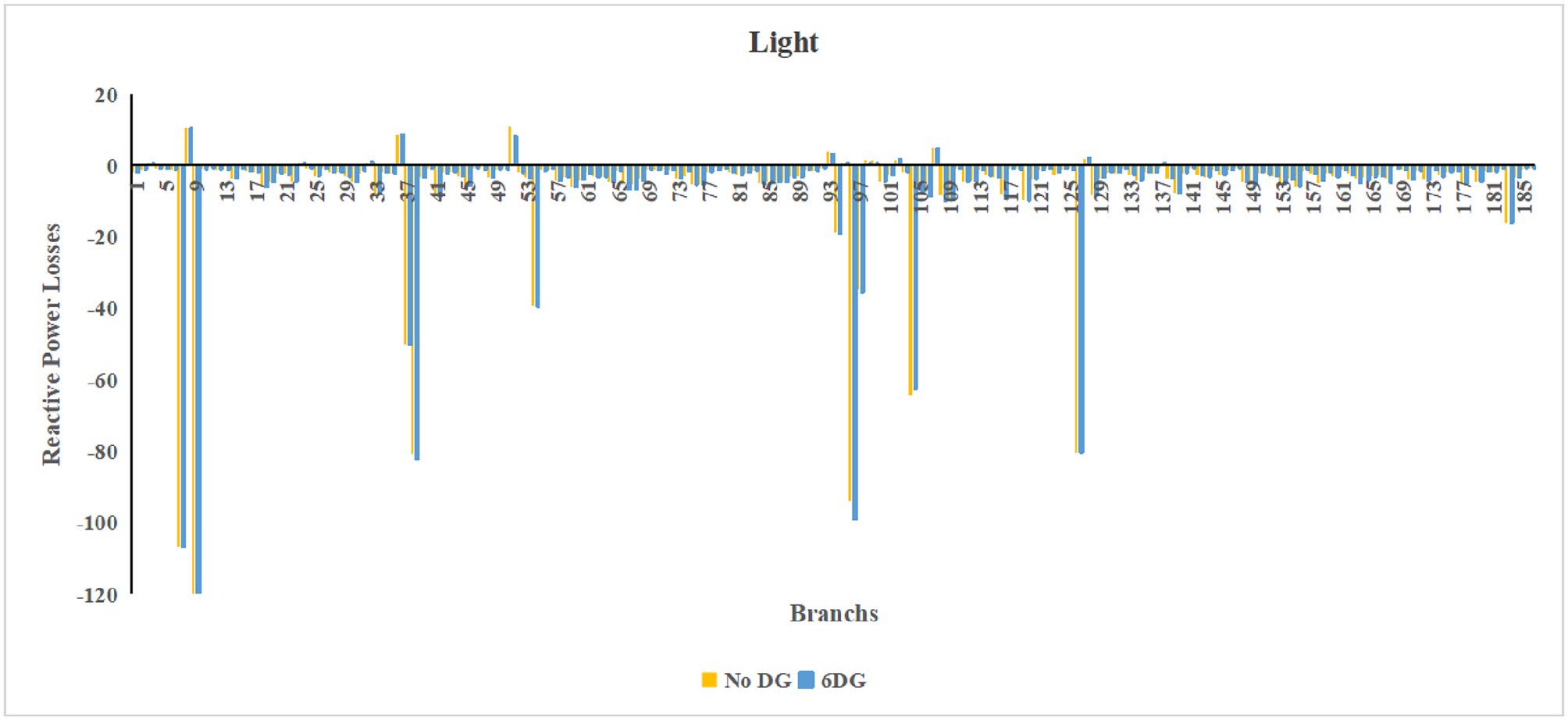
Reactive power losses across all branches in light load condition.

**Fig. 62:**
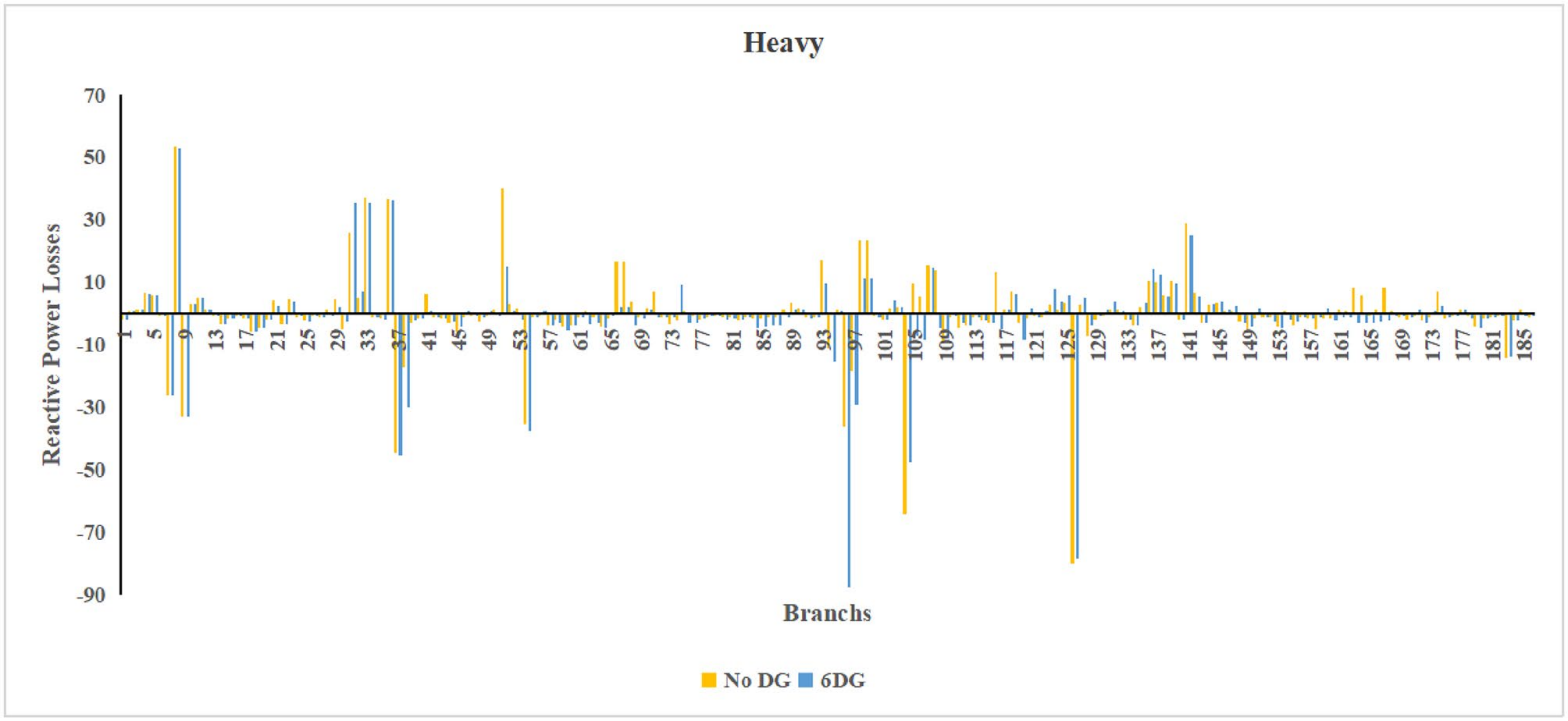
Reactive power losses across all branches in heavy load condition.

A similar trend is observed for reactive power losses in Figures 60, 61 and 62. The distributed generation effectively supplied a portion of the local reactive demand, reducing the reactive current component flowing through the transmission lines. This drastically minimized the reactive voltage drops across the network and alleviated the burden on the central synchronous generators. This local relief mechanism is a primary driver behind the global performance enhancement of the IEEE 118-bus transmission system.

## Conclusion

The current research paper develops a complete optimization methodology that uses the Dingo Optimization Algorithm (DOA) in addressing the challenging issues associated with the DG sizing and allocation problem. The use of the weighted sum sizing technique (0.2 for light, 0.6 for normal, and 0.2 for heavy) successfully closes the difference between theoretical single scenario optimization and dynamic multiple scenarios in real-world situations.

Deployment of DOA in both radial and meshed systems shows how scalable and robust the algorithm can be. In tests on the 33-bus system, where comparisons were made against conventional algorithms, DOA showed better convergence behavior by lowering the active power loss by 81.63%, while raising the lowest critical voltage value from 0.9134 p.u. to 0.9732 p.u.

From the 118-bus system evaluation, it is also evident that the spatial placement of the DG units is essential. The case involved using three DG units comprising only PV units at a power factor of 0.95, which resulted in a loss reduction of 16.24%, but their impact was not adequate for the extensive and interconnected network. However, when the network grew from three buses to six buses with a hybrid of PV-Wind units, the efficiency increased to 23.98%, which corresponds to savings of more than 53 MW, reducing the maximum FVSI to 0.2817.

This research brings to attention a key weakness in operations that is caused by uncontrolled injection of renewable energy during low-load hours, which causes an undesired reverse flow of power. The implementation of the Dynamic Dispatch Factor of 0.3 resulted in transforming a considerable threat to performance (a loss of −42.07%) into a benefit of 12.61%.

From the analysis, it is evident that the DOA optimization technique is both flexible and effective, making it suitable for the optimization of power grid designs in modern times with the ability to tackle the physical constraints of hybrid renewable energy systems. The next step in the research should be to explore how BESS can be employed to tap energy during light load periods.

## Data Availability

The datasets generated during and/or analysed during the current study are available from the corresponding author on reasonable request. The data used for the IEEE 33-bus and IEEE 118-bus systems are available in the References^27,28^.
